# Expressing 2-keto acid pathway enzymes significantly increases photosynthetic isobutanol production

**DOI:** 10.1186/s12934-022-01738-z

**Published:** 2022-02-01

**Authors:** Hao Xie, Peter Lindblad

**Affiliations:** Microbial Chemistry, Department of Chemistry-Ångström Laboratory, Uppsala University, Box 523, 75120 Uppsala, Sweden

**Keywords:** *Synechocystis* PCC 6803, Isobutanol production, 2-Keto acid pathway, α-Ketoisovalerate decarboxylase, Metabolic engineering

## Abstract

**Background:**

Cyanobacteria, photosynthetic microorganisms, are promising green cell factories for chemical production, including biofuels. Isobutanol, a four-carbon alcohol, is considered as a superior candidate as a biofuel for its high energy density with suitable chemical and physical characteristics. The unicellular cyanobacterium *Synechocystis* PCC 6803 has been successfully engineered for photosynthetic isobutanol production from CO_2_ and solar energy in a direct process.

**Results:**

Heterologous expression of α-ketoisovalerate decarboxylase (Kivd^S286T^) is sufficient for isobutanol synthesis via the 2-keto acid pathway in *Synechocystis*. With additional expression of acetolactate synthase (AlsS), acetohydroxy-acid isomeroreductase (IlvC), dihydroxy-acid dehydratase (IlvD), and alcohol dehydrogenase (Slr1192^OP^), the *Synechocystis* strain HX42, with a functional 2-keto acid pathway, showed enhanced isobutanol production reaching 98 mg L^−1^ in short-term screening experiments. Through modulating *kivd*^*S286T*^ copy numbers as well as the composition of the 5′-region, a final *Synechocystis* strain HX47 with three copies of *kivd*^*S286T*^ showed a significantly improved isobutanol production of 144 mg L^−1^, an 177% increase compared to the previously reported best producing strain under identical conditions.

**Conclusions:**

This work demonstrates the feasibility to express heterologous genes with a combination of self-replicating plasmid-based system and genome-based system in *Synechocystis* cells. Obtained isobutanol-producing *Synechocystis* strains form the base for further investigation of continuous, long-term-photosynthetic isobutanol production from solar energy and carbon dioxide.

**Graphic abstract:**

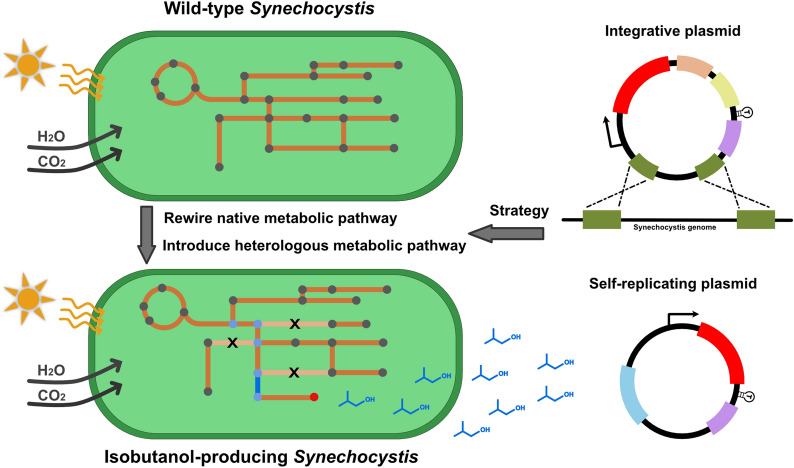

**Supplementary Information:**

The online version contains supplementary material available at 10.1186/s12934-022-01738-z.

## Introduction

As an effective approach to alleviate the increased demand of energy and the concerns of global climate change caused by CO_2_ emissions, there is a great urgency to develop biofuels as new energy carriers to replace presently used fossil resources [1]. Isobutanol, a four-carbon alcohol, is one of the preferred candidates as biofuels due to its superior characteristics, such as high combustion power, low hygroscopicity, and low water solubility [2]. Isobutanol has a similar energy density as 1-butanol but a higher octane number than 1-butanol, (research, motor and pump octane numbers of 114, 94 and 104 compared to 96, 78 and 87, respectively), making it preferred for blending into gasoline and more readily upgraded to renewable jet fuel blendstock [3]. Additionally, isobutanol is an important bulk chemical with many direct and indirect applications, including but not limited to be used in plastics, coating, and pharmaceutical industries [3–5].

**Fig. 1:**
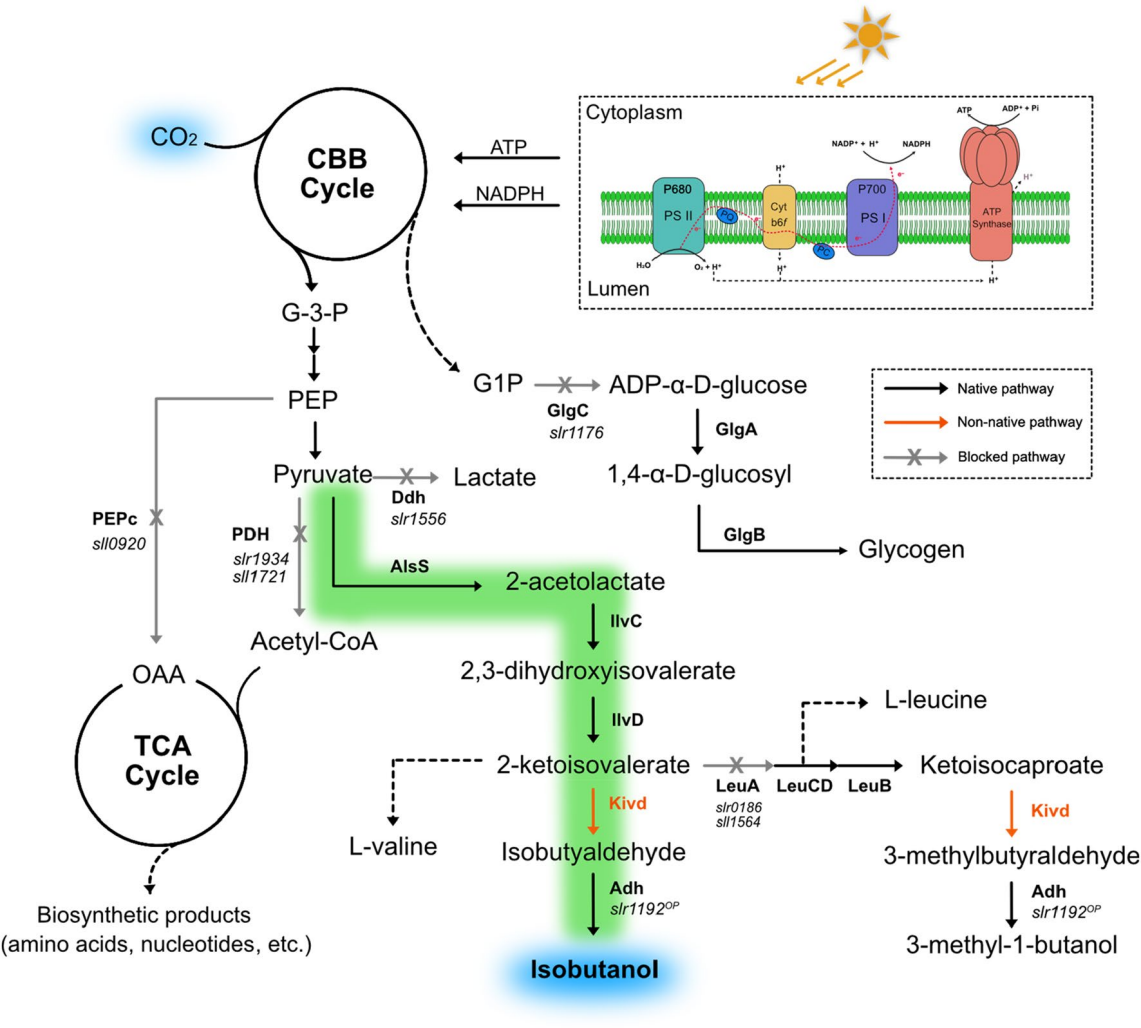
Overview of isobutanol biosynthesis pathway in *Synechocystis* PCC 6803 and metabolic engineering strategies examined in this study. Photosynthesis generates ATP and reducing equivalents in the form of NADPH, which are used for carbon dioxide fixation via the CBB (Calvin-Benson-Bassham) cycle. Foreign enzymes are in red font while the native enzymes are in black font. Abbreviations for enzymes: AlsS, acetolactate synthase (*Bacillus subtilis*); IlvC, acetohydroxy-acid isomeroreductase (*Escherichia coli*); IlvD, dihydroxy-acid dehydratase (*E. coli*); Kivd, α-ketoisovalerate decarboxylase (*Lactococcus lactis*); Slr1192^OP^, codon optimized alcohol dehydrogenase (*Synechocystis*); PEPc, phosphoenolpyruvate carboxylase; Ddh, d-lactate dehydrogenase; GlgC: glucose-1-phosphate adenylyltransferase; GlgA, glycogen synthase; GlgB, 1,4-α-glucan branching enzyme; LeuA, 2-isopropylmalate synthase; LeuCD, 3-isopropylmalate dehydratase; LeuB, 3-isopropylmalate dehydrogenase; PDH, pyruvate dehydrogenase E1 component. Abbreviations for metabolites: G-3-P, glyceraldehyde-3-phosphate; G1P, glucose-1-phosphate; PEP, phosphoenolpyruvate; OAA, oxaloacetate. Black lines indicate native pathways; red lines indicate non-native pathways; grey lines indicate the blocked pathways

Biological isobutanol production was first demonstrated in *Escherichia coli* through the implementation of a non-fermentative pathway, the so-called 2-keto acid pathway [2]. The 2-keto acid pathway, using pyruvate as starting metabolite, involves five enzymes: acetolactate synthase (AlsS), acetohydroxy-acid isomeroreductase (IlvC), dihydroxy-acid dehydrase (IlvD), α-ketoisovalerate decarboxylase (Kivd), and alcohol dehydrogenase (Adh) (Fig. 1). As a key enzyme of the 2-keto acid pathway, Kivd decarboxylates 2-ketoisovalerate, an intermediate of the valine synthesis pathway, into isobutyraldehyde, which is further converted to isobutanol by Adh. Later on, the same strategy was extensively applied in various microorganisms, including *Saccharomyces cerevisiae* [6]*, Corynebacterium glutamicum* [7], *Ralstonia eutropha* [8], *Clostridium cellulolyticum* [9], *Bacillus subtilis* [10] as well as cyanobacteria [11–14].

Different from heterotrophic microorganisms, cyanobacteria are photoautotrophic microorganisms capable of performing photosynthesis using water and carbon dioxide as substrates and sunlight as energy source. *Synechococcus elongatus* PCC 7942 (hereafter *Synechococcus)* was the first cyanobacterium being engineered for isobutanol synthesis, by expressing AlsS from *B. subtilis*, IlvC and IlvD from *E. coli*, Kivd from *Lactococcus lactis*, and Adh (YqhD) from *E. coli* [11]. In the same study, increased isobutanol production was observed after overexpressing ribulose 1,5-bisphosphate carboxylase/oxygenase (Rubisco), a rate limiting enzyme in the Calvin Benson Bassham (CBB) cycle [11]. Following the first proof-of-concept study to develop cyanobacteria as biocatalysts to produce isobutanol directly from solar energy and CO_2_, *Synechocystis* PCC 6803 (hereafter *Synechocystis*), another model cyanobacterium, was demonstrated to have the capability for isobutanol synthesis with a single heterologous expression of Kivd from *L. lactis*, with 3-methyl-1-butanol (3M1B) produced as minor by-product [14]. Moreover, Kivd was identified as one bottleneck in the isobutanol synthesis pathway [14]. In a following study, an engineered Kivd^S286T^ with a single amino acid replacement resulted in higher isobutanol production as well as higher isobutanol-to-3M1B molar ratio than the original Kivd [15]. Apart from genomic modifications, cultivation condition optimization also plays a significant role in further enhancement of isobutanol production in *Synechocystis*. By stabilizing the pH to between 7 and 8 using HCl titration, an increase of isobutanol production of 273% was observed after 10-day cultivation [12].

As established previously [12], two distinct cultivation methods, a short-term screening experiment and a long-term milking experiment, have been employed for examining isobutanol production in *Synechocystis*. The short-term screening experiment is used to compare different engineered *Synechocystis* strains and aims to identify a strain with the best performance of isobutanol synthesis, while the main purpose of a long-term milking experiment is to explore the capacity of isobutanol production under a given environmental condition of a selected *Synechocystis* strain.

In the present study, short-term screening experiments were used to examine isobutanol production in engineered *Synechocystis* strains. Our previous best isobutanol producing strain pEEK2-ST, with Kivd^S286T^ expressed from a self-replicating plasmid, producing 52 mg isobutanol L^−1^ in short-term screening experiments [12], was used as parental strain. Different strategies were explored to further enhance isobutanol production of *Synechocystis* cells. Two protein expression systems, a self-replicating plasmid-based system (selected gene placed on self-replicating plasmid) and a genome-based system (selected gene integrated into the chromosome), were introduced simultaneously into the *Synechocystis* cells. Further optimization of the 2-keto acid pathway was performed by overexpressing selected enzymes from the valine synthesis pathway, with the aim to increase the carbon flux towards the 2-keto acid pathway. Similar to what has been observed in *Synechococcus* [11], for the first time, an engineered *Synechocystis* cell with a complete 2-keto acid pathway was successfully generated, with a simultaneous increase in isobutanol production. On the other hand, both transcription and translation of *kivd*^*S286T*^ were tuned for enhanced protein expression levels, further contributing to improvement of isobutanol production in *Synechocystis* cells. Moreover, gene dosage of *kivd*^*S286T*^ was manipulated through multiple integrations into different sites of the chromosome and on a self-replicating plasmid, which is confirmed to be positively correlated to isobutanol production of *Synechocystis* cells.

## Materials and methods

### Strains used in cloning, transformation and conjugation

*Escherichia coli* strains DH5α-Z1 (Invitrogen) and T7 Express (NEB) were used for cloning. The cells were grown at 37 °C in lysogeny broth (LB) medium (Sigma-Aldrich) supplemented with appropriate antibiotic(s). The final concentration used for kanamycin (Thermo Fisher Scientific), chloramphenicol (Sigma-Aldrich) and spectinomycin (AppliChem) were 50 μg mL^−1^, 35 μg mL^−1^, and 50 μg mL^−1^, respectively. *E. coli* HB101 helper cell with plasmid pRL443-Amp^R^ was used for conjugation. The glucose-tolerant *Synechocystis* PCC 6803 strain was used throughout this study. *Synechocystis* seed cultures were maintained under 30 μmol photons m^−2^ s^−1^ at 30 °C in BG11 medium [16].

### Plasmid construction

All heterologous genes and endogenous genes were codon optimized and synthesized by GenScript and are listed in Additional file 1: Table S1. Homologous recombination regions were amplified using specific primers from wild-type *Synechocystis* genome using Phusion Polymerase (Thermo Scientific). P*psbA2*, RBS*, Terminator BBa_B0015 were amplified from pEERM1 [17]. P*trc*BCD and P*trc*RiboJ promotor fragments were amplified from pEEC series of vectors [18]. All integrative vectors were based on the recently reported pEERM series of vectors [17]. The homologous recombination regions are the around 1000 bp upstream sequence and the around 1000 bp downstream sequence of the integrated sites in the chromosome. BglΙΙ, EcoRΙ, XbaΙ, SpeΙ, PstΙ, BamHI and SalΙ restriction enzymes were used to assemble all plasmids in this study. All enzymatic digestions were performed with FastDigest enzymes (Thermo Scientific). Ligation reactions were performed using the Quick Ligation kit from New England Biolabs (NEB). The plasmids constructed in this study are listed in Additional file 1: Table S2. The Primers used for plasmids construction in this study are listed in Additional file 1: Table S3.

### Transformation methods for *Synechocystis*

#### Natural transformation

*Synechocystis* cells were grown to mid-log phase (OD_750_ = 0.5–1.2) in liquid BG11 medium. Then cells were collected by centrifuging at 7200 rpm/5000×*g* for 5 min and washed twice with fresh liquid BG11 medium without antibiotics, and finally resuspended in fresh BG11 medium at a density of 1 × 10^9^ cells mL^−1^. A total of 400 μL of concentrated *Synechocystis* cells were mixed with 4 μg of plasmid DNA. After incubation under illumination of 50 μmol photons m^−2^ s^−1^ at 30 °C for 4–5 h, the cells were spread onto nitrocellulose membranes on BG11 agar plates without antibiotics for another 24 h incubation. For colony selection and maintenance, the membranes were transferred onto new BG11 agar plates with appropriate antibiotic(s), and incubated under the same conditions as before. Isolated single colony was streaked onto new BG11 agar plates with appropriate antibiotics, and were analyzed by colony PCR using gene specific primers (Additional file 1: Table S3) and DreamTaq DNA polymerase (Thermo Scientific). The verified transformants with expression cassette integrated into the genome were then inoculated into 6-well plate (SARSTEDT) in BG11 medium with corresponding antibiotic(s), and propagated until fully segregation. The segregation of each strain was examined by PCR using gene specific primers (Additional file 1: Table S3) and DreamTaq DNA polymerase (Thermo Scientific) on the extracted genomic DNA, which is prepared based on methods described in [19].

#### Conjugation

*Synechocystis* cells were grown to mid-log phase (OD_750_ = 0.5–1.2) in liquid BG11 medium. *E. coli* HB101 conjugative cells with the plasmid pRL443-Amp^R^ and *E. coli* cells with cargo plasmid were grown overnight at 37 °C. One milliliter of *E. coli* and *Synechocystis* cells were centrifuged (7200 rpm/5000×*g* for 10 min) at room temperature. The *E. coli* cargo and conjugative cells were resuspended in 500 µL of LB without antibiotics, combined with each other and washed once with fresh medium before being resuspended in 500 µL of LB without antibiotics. The *Synechocystis* cells was washed twice with fresh medium and then resuspended in 500 µL of fresh BG11 without antibiotics. A mixture of cargo cells (250 μL), conjugative cells (250 μL) and *Synechocystis* cells (100 μL) was incubated under illumination of 50 μmol photons m^−2^ s^−1^ at 30 °C for 1.5 h, before being spread onto membranes on BG11 agar plates without antibiotics for 24 h. For colony selection and maintenance, the membranes were transferred onto new BG11 agar plates with appropriate antibiotic(s), and incubated under the same conditions as before. Positive colonies were verified by colony PCR using gene specific primers (Additional file 1: Table S3) and DreamTaq DNA polymerase (Thermo Scientific). All engineered *Synechocystis* strains generated in this study are summarized in Table 1.

**Table 1:** *Synechocystis* PCC 6803 strains used in this study

Strain	Relevant genotypes^a^	Reference
WT	Wild-type *Synechocystis* PCC 6803	[14]
pEEK2-ST	pEEK2-(P*trc*BCD-***kivd***^***S286T***^-T)-Km^R^	[15]
HX0	*Δddh*::Cm^R^, pEEK2-(P*trc*BCD-***kivd***^***S286T***^-T)-Km^R^	This study
HX1	*Δddh*::(P*psbA2*-***kivd***^***S286T***^-T)-Cm^R^, pEEK2-(P*trc*BCD-***kivd***^***S286T***^-T)-Km^R^	This study
HX2	*Δddh*::(P*trc*BCD-***kivd***^***S286T***^-T)-Cm^R^, pEEK2-(P*trc*BCD-***kivd***^***S286T***^-T)-Km^R^	This study
HX3	*Δddh*::(P*trc*RiboJ-***kivd***^***S286T***^-T)-Cm^R^, pEEK2-(P*trc*BCD-***kivd***^***S286T***^-T)-Km^R^	This study
HX5	*Δddh*::(P*psbA2*-***alsS***-T)-Cm^R^, pEEK2-(P*trc*BCD-***kivd***^***S286T***^-T)-Km^R^	This study
HX6	*Δddh*::(P*trc*BCD-***alsS***-T)-Cm^R^, pEEK2-(P*trc*BCD-***kivd***^***S286T***^-T)-Km^R^	This study
HX7	*Δddh*::(P*trc*BCD-***kivd***^***S286T***^-***alsS***-T)-Cm^R^, pEEK2-(P*trc*BCD-***kivd***^***S286T***^-T)-Km^R^	This study
HX8	*Δddh*::(P*trc*BCD-***ilvD***-***alsS***-T)-Cm^R^, pEEK2-(P*trc*BCD-***kivd***^***S286T***^-T)-Km^R^	This study
HX9	*Δddh*::(P*trc*BCD-***slr1192***^***OP***^-***alsS***-T)-Cm^R^, pEEK2-(P*trc*BCD-***kivd***^***S286T***^-T)-Km^R^	This study
HX15	*Δddh*::(P*trc*BCD-***kivd***^***S286T***^-T)-Cm^R^, *Δslr0168*::(P*trc*BCD-***kivd***^***S286T***^-T)-Sp^R^, pEEK2-(P*trc*BCD-***kivd***^***S286T***^-T)-Km^R^	This study
HX16	*Δddh*::(P*trc*BCD-***kivd***^***S286T***^-T)-Cm^R^, *Δslr0168*::Sp^R^, pEEK2-(P*trc*BCD-***kivd***^***S286T***^-T)-Km^R^	This study
HX17	*Δddh*::Cm^R^, *Δslr0168*::Sp^R^, pEEK2-(P*trc*BCD-***kivd***^***S286T***^-T)-Km^R^	This study
HX28	*Δddh*::(P*trc*BCD-***kivd***^***S286T***^-T)-Cm^R^, *Δslr0168*::(P*trc*BCD-***kivd***^***S286T***^-T)-Sp^R^, pEEK2-(P*trc*BCD-***kivd***^***S286T***^-T)-Km^R^	This study
HX29	*Δddh*::(P*trc*BCD-***kivd***^***S286T***^-T)-Cm^R^, *Δsll1564*::(P*trc*BCD-***kivd***^***S286T***^-T)-Sp^R^, pEEK2-(P*trc*BCD-***kivd***^***S286T***^-T)-Km^R^	This study
HX39	*Δddh*::(P*trc*BCD-***kivd***^***S286T***^-T)-Cm^R^, *ΔPEPc*::(P*trc*BCD-***kivd***^***S286T***^-T)-Sp^R^, pEEK2-(P*trc*BCD-***kivd***^***S286T***^-T)-Km^R^	This study
HX40	*Δddh*::(P*trc*BCD-***kivd***^***S286T***^-***slr1192***^***OP***^-T)-Cm^R^, pEEK2-(P*trc*BCD-***kivd***^***S286T***^-T)-Km^R^	This study
HX42	*Δddh*::(P*trc*BCD-***slr1192***^***OP***^-***alsS***-T)-Cm^R^, *Δslr0168*::(P*trc*BCD-***ilvC***-***ilvD***-T)-Sp^R^, pEEK2-(P*trc*BCD-***kivd***^***S286T***^-T)-Km^R^	This study
HX43	*Δddh*::(P*trc*BCD-***kivd***^***S286T***^-***slr1192***^***OP***^-***alsS***-T)-Cm^R^, *Δslr0168*::(P*psbA2*-***ilvC***-***ilvD***-T)-Sp^R^, pEEK2-(P*trc*BCD-***kivd***^***S286T***^-T)-Km^R^	This study
HX44	*Δddh*::(P*trc*BCD-***kivd***^***S286T***^-T)-Cm^R^, *Δslr1934*::(P*trc*BCD-***kivd***^***S286T***^-T)-Sp^R^, pEEK2-(P*trc*BCD-***kivd***^***S286T***^-T)-Km^R^	This study
HX45	*Δddh*::(P*trc*BCD-***kivd***^***S286T***^-T)-Cm^R^, *Δslr0186*::(P*trc*BCD-***kivd***^***S286T***^-T)-Sp^R^, pEEK2-(P*trc*BCD-***kivd***^***S286T***^-T)-Km^R^	This study
HX46	*Δddh*::(P*trc*BCD-***kivd***^***S286T***^-T)-Cm^R^, *Δsll1721*::(P*trc*BCD-***kivd***^***S286T***^-T)-Sp^R^, pEEK2-(P*trc*BCD-***kivd***^***S286T***^-T)-Km^R^	This study
HX47	*Δddh*::(P*trc*BCD-***kivd***^***S286T***^-T)-Cm^R^, *ΔglgC*::(P*trc*BCD-***kivd***^***S286T***^-T)-Sp^R^, pEEK2-(P*trc*BCD-***kivd***^***S286T***^-T)-Km^R^	This study

Expressed genes in bold

^a^Km^R^, Kanamycin resistance cassette; Sp^R^, spectinomycin resistance cassette; Cm^R^: chloramphenicol resistance cassette; T, Terminator BBa_B0015

### Cultivation condition of short-term screening experiment

Seed cultures were grown under 30 μmol photons m^−2^ s^−1^ at 30 °C in BG11 with appropriate antibiotic(s) in 100 mL Erlenmeyer flasks (VWR) until OD_750_ = 1.5–2.0. The seed cultures were then used to inoculate 25 mL experimental cultures to OD_750_ = 0.1 in BioLite 25 cm^2^ plug-sealed tissue culture flasks (Thermo Fisher Scientific). The medium used for experimental culture was BG11 with addition of 50 mM NaHCO_3_ (Sigma-Aldrich) and appropriate antibiotic(s) (final concentration: chloramphenicol 20 μg mL^−1^, spectinomycin 50 μg mL^−1^, and kanamycin 50 μg mL^−1^). All experimental cultures were prepared in triplicate. The flasks were shaken horizontally at 120 rpm, under 50 μmol photons m^−2^ s^−1^ at 30 °C. Two milliliter of culture was sampled from each flask every second day for measurements and 2 mL of fresh BG11 medium with addition of 500 mM NaHCO_3_ (Sigma-Aldrich) and appropriate antibiotic(s) were added back. The cultivation was terminated when the isobutanol production in the culture started to decrease.

### RNA isolation and semi-quantitative reverse transcript PCR (RT-PCR)

*Synechocystis* cells were harvested at OD_750_ = 0.3–0.7. Total RNA was isolated from cell culture using RTI Reagent (Sigma-Aldrich) according to the manufacturer’s instructions. The RNA concentration was measured using a Nanodrop™ 2000 spectrophotometer (Thermo Fisher Scientific). 1 μg of RNA and qScript™ cDNA Synthesis Kit were used for cDNA synthesis. 23S RNA was used as control.

### Crude protein extraction and SDS-PAGE/Western-immunoblot

Proteins were extracted from cell cultures with OD_750_ = 2.0, and 5 mL of cell culture was harvested by centrifugation at 5000 rpm/4500×*g* for 10 min at room temperature. Obtained cell pellets were washed with 2 mL of PBS and centrifuged again at 5000 rpm/2400×*g* for 10 min at room temperature. The pellets were resuspended in 200 μL of PBS and were frozen in − 80 °C for 10 min followed by heating at 37 °C for 10 min. For the following steps, the samples were kept on ice. Four microliters of 50X Protease Arrest (GBioscience) and acid-washed glass beads (425–600 μm diameter, Sigma-Aldrich) were mixed with cells, which were disrupted by using the Precellys-24 Beadbeater (Bertin Instruments) with 5000 rpm program 4 × 30 s. After adding 100 μL of PBS, the total lysates were centrifuged twice at 1976 rpm/1000×*g*, 4 °C, 30 s, to obtain a transparent supernatant containing soluble proteins. The protein concentrations were determined by the DC protein assay (Bio-Rad).

Five micrograms (Strep-tagged proteins) and 20 μg (His-tagged and Flag-tagged proteins) of soluble proteins were loaded and separated on sodium dodecyl sulfate polyacrylamide gel electrophoresis (SDS-PAGE), using Mini-PROTEAN TGX™ gels (Bio-Rad), and transferred to PVDF membranes (Bio-Rad). Proteins were detected using anti-Strep-tag (abcam, ab76949), anti-Flag-tag (Sigma-Aldrich, F3165) and anti-His-tag (GenScript, A00186) primary antibodies and HRP-conjugated secondary antibodies: goat-anti-rabbit lgG (Bio-Rad, 972-4446) for Strep-tag; rabbit-anti-mouse lgG (Agrisera, AS101114) for both Flag-tag and His-tag. Bands were detected using the Clarity ECL substrate (Bio-Rad) and quantified using Quantity One Software (Bio-Rad).

### Optical density measurement and isobutanol extraction

Cell growth was monitored by measuring optical density at 750 nm (OD_750_) of each cell culture. The absorbance at 750 nm was measured every day for 200 μL cell culture in 96-well plate (SARSTEDT) using a microplate reader (HIDEX, Plate Chameleon).

Every second day, 2 mL of cell culture were sampled from each flask and centrifuged at 5000 rpm/2400×*g* for 10 min. Then, 1305 μL of supernatant were transferred into a 15 mL screw cap tube and mixed with 45 μL of 3000 mg L^−1^ internal standard 1-pentanol (Sigma-Aldrich) and 450 μL of extraction solvent dichloromethane (DCM, Sigma-Aldrich). The mixture was shaken on Multi-tube Vortexer VX-2500 (VWR) at maximal speed for 5 min, followed by centrifugation at 5000 rpm/4500×*g*, 4 °C, for 10 min. DCM phase was then transferred into 1.5 mL clear glass gas chromatography vials (VWR) for gas chromatography analysis.

### Isobutanol quantification by gas chromatography

A detailed protocol for isobutanol quantification has been described [14]. In short, the extracted samples were analyzed on a PerkinElmer GC 580 system equipped with a flame ionization detector (FID) and an Elite-WAX Polyethylene Glycol Series Capillary column, 30 m × 0.25 mm × 0.25 μm (PerkinElmer). Nitrogen was the carrier gas with a rate of 10 mL min^−1^. The temperatures of injector and detector were 220 °C and 240 °C, respectively. Obtained GC results were analyzed using TotalChrom Navigator version 6.3.2.

## Results and discussion

### Expressing AlsS has an effect on isobutanol biosynthesis

Acetolactate synthase (AlsS), catalyzing condensation of pyruvate into 2-acetolactate, is the first enzyme of the 2-keto acid pathway, which plays a vital role in converting more carbon from the central metabolite pyruvate towards isobutanol synthesis. It has been shown that overexpression of *alsS* contributed to a 1.8-fold improvement of isobutanol production in *B. subtilis* [10]. On the other hand, AlsS functions not only as acetolactate synthase to condense two pyruvate molecules, but also it was experimentally verified that it functions as α-ketoisovalerate decarboxylase [20]. Previously, α-ketoisovalerate decarboxylase was identified as the bottleneck in the isobutanol synthesis pathway in *Synechocystis* cells [12]. Here, the effects of expressing AlsS on isobutanol production was explored in isobutanol-producing *Synechocystis* strains.

**Fig. 2:**
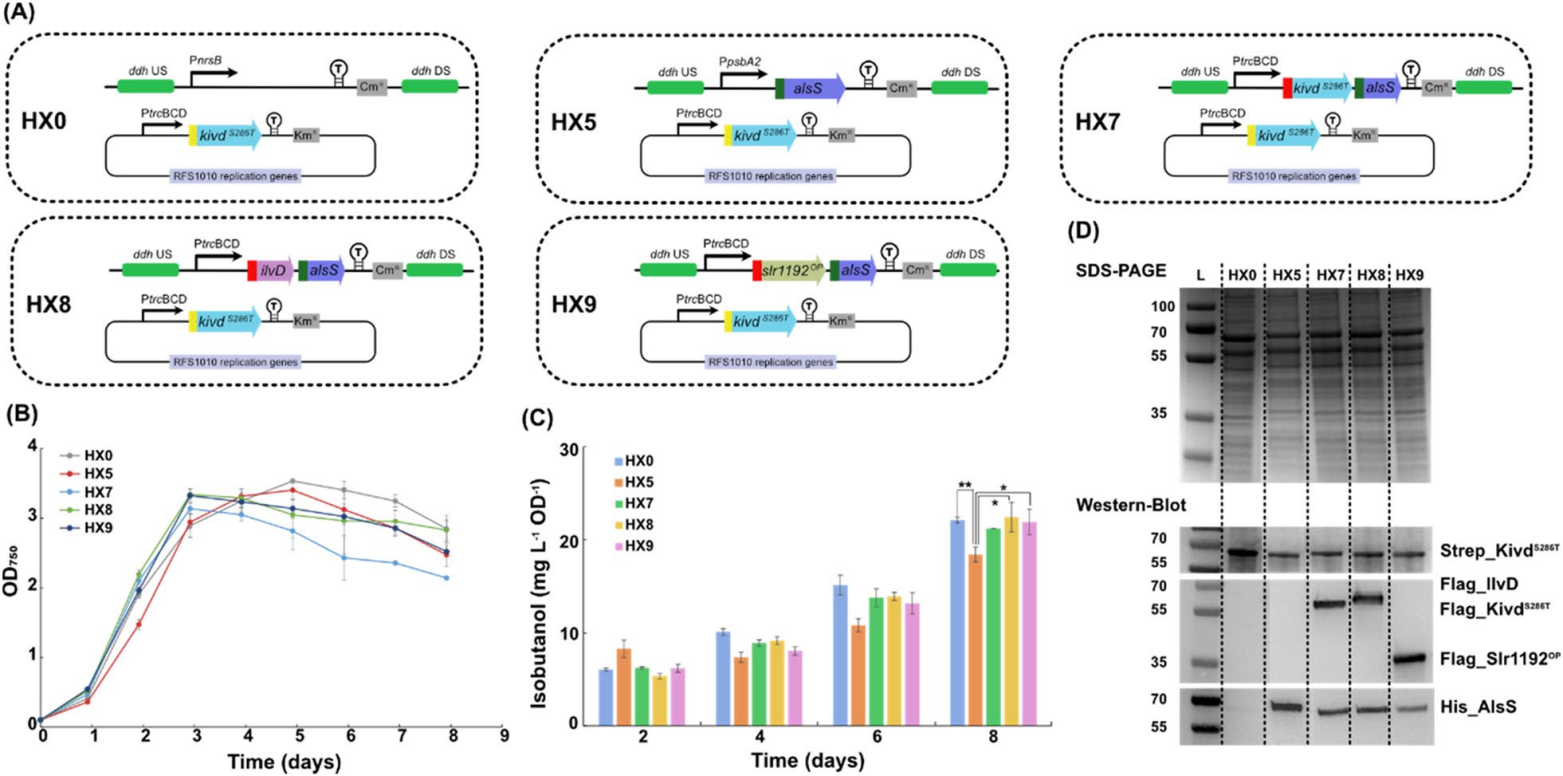
Schematic overview of genetic constructs used and comparison of growth, protein levels (SDS-PAGE/Western-immunoblot) and isobutanol production per cell in engineered *Synechocystis* PCC 6803 strains HX0, HX5, HX7, HX8, and HX9. **A** Schematic presentation of the genetic constructs in the engineered strains. *kivd*^*S286T*^: encodes α-ketoisovalerate decarboxylase (*Lactococcus lactis*); *alsS*: encodes acetolactate synthase (*Bacillus subtilis*); *ilvD*: encodes dihydroxy-acid dehydratase (*Escherichia coli*); *slr1192*^*OP*^: encodes codon optimized alcohol dehydrogenase (*Synechocystis*). Kivd^S286T^ expressed on self-replicating plasmid was Strep-tagged at the N-terminal; Kivd^S286T^, IlvD*, and* Slr1192^OP^ expressed in the *ddh* (*slr1556*) site of chromosome was Flag-tagged at the N-terminal; AlsS expressed in the *ddh* (*slr1556*) site of chromosome was His-tagged at the N-terminal. **B** Growth curves of the engineered strains during 8-day cultivation. **C** Isobutanol production per cell at day 2, 4, 6, and 8 of indicated strains. **D** SDS-PAGE (top) and Western-immunoblot (bottom). Each lane represents result from respective strain. L: ladder (in kDa). For SDS-PAGE, 20 μg of total soluble protein were loaded for each strain. For Western-immunoblot, 5 μg, 20 μg, and 20 μg of total soluble protein were loaded for each strain to detect Strep-tagged, Flag-tagged, and His-tagged protein, respectively. Results are the mean of three biological replicates, each with three technical replicates. Error bars represent standard deviation. Asterisk represents significant difference between different strains (one-way ANOVA, *p < 0.05, **p < 0.005)

It has been reported to be challenging to get successful transformants containing AlsS, not only in *Synechocystis* [12], but also in *C. cellulolyticum* [9], possibly due to the high activity of AlsS [9]. In the present study, instead of being placed on self-replicating plasmid, *alsS* was integrated in the chromosome of *Synechocystis* for expression. To increase the probability of successfully generating *Synechocystis* transformants containing AlsS, AlsS expression was controlled by a selection of promoters with varied strength and organization of gene orders within the operon. HX0 was generated as a control strain, with chloramphenicol resistance (Cm^R^) cassette integrated into the *ddh* site and *Strep-kivd*^*S286T*^ placed on a self-replicating plasmid (Fig. 2A). In HX5, P*psbA2* was used to drive *alsS* expression (Fig. 2A). As expected, when changing from P*psbA2* to P*trc*BCD, AlsS could not be detected by Western-immunoblot (Additional file 1: Fig. S1). Spontaneous mutation(s) of AlsS may occur due to its high activity [9]. In addition, *alsS* was expressed with either *Flag-kivd*^*S286T*^, *ilvD* or *slr1192*^*OP*^ as an operon, to generate strains HX7, HX8, and HX9 (Fig. 2A). In all strains, apart from integrating selected genes into the *ddh* site of chromosome, *Strep-kivd*^*S286T*^ was placed on self-replicating plasmid for expression (Fig. 2A). AlsS was successfully detected in all four engineered strains by Western-immunoblot (Fig. 2D). This is the first report demonstrating that *alsS* was integrated into *Synechocystis* chromosome, and its expression was successfully confirmed by Western-immunoblot (Fig. 2D). Unexpectedly, none of the four engineered strains produced significantly higher isobutanol than the control strain HX0 (Fig. 2C).

With almost the same isobutanol production per cell achieved by strains HX0, 7, 8 and 9 (Fig. 2C), it is interesting to observe that the Strep_Kivd^S286T^ expression level was not consistent in the four strains, and a significant decrease of Strep-Kivd^S286T^ protein level was detected in strains HX7, 8 and 9 (Fig. 2D; Additional file 1: Table S4).

The broad host range RSF1010 replicon-based self-replicating plasmid is widely used when demonstrating biofuel production in cyanobacteria [18, 21, 22]. However, this is the first study expressing genes with both a self-replicating plasmid-based system and a genome-based system in *Synechocystis* cells. Interestingly, self-replicating plasmid-based system may not be as stable as expected. When a heterologous gene is integrated in the chromosome, the expression level of a heterologous gene on a self-replicating plasmid is affected dramatically (Fig. 2D; Additional file 1: Table S4). In our previous study, it was observed that isobutanol production was positively correlated with Kivd^S286T^ expression level [12]. However, in the present study, similar isobutanol production was observed (Fig. 2C), though less Strep-Kivd^S286T^ is expressed in the engineered strains HX7-9 compared to the control strain HX0 (Fig. 2D; Additional file 1: Table S4). Therefore, additional expression of AlsS may make up for the decreased isobutanol production as a result of the decreased expression level of Strep-Kivd^S286T^, enabling the engineered strains to reach similar isobutanol production per cell as the control strain. On the other hand, different from other engineered strains, HX5 grew slower between days 0 and 3 (Fig. 2B; Additional file 1: Fig. S2) and produced less isobutanol per cell than control strain HX0 (Fig. 2C), which may be explained by the significantly decreased expression of Kivd^S286T^ (Fig. 2D; Additional file 1: Table S4). When compared with strains HX8-9, HX5 produced less isobutanol (Fig. 2C), although a higher expression level of AlsS was observed in HX5 (Fig. 2D; Additional file 1: Table S4), indicating that IlvD and Slr1192^OP^ are important enzymes for further enhancement of isobutanol production in *Synechocystis*.

### Complete 2-keto acid pathway integration enhances isobutanol production

**Fig. 3:**
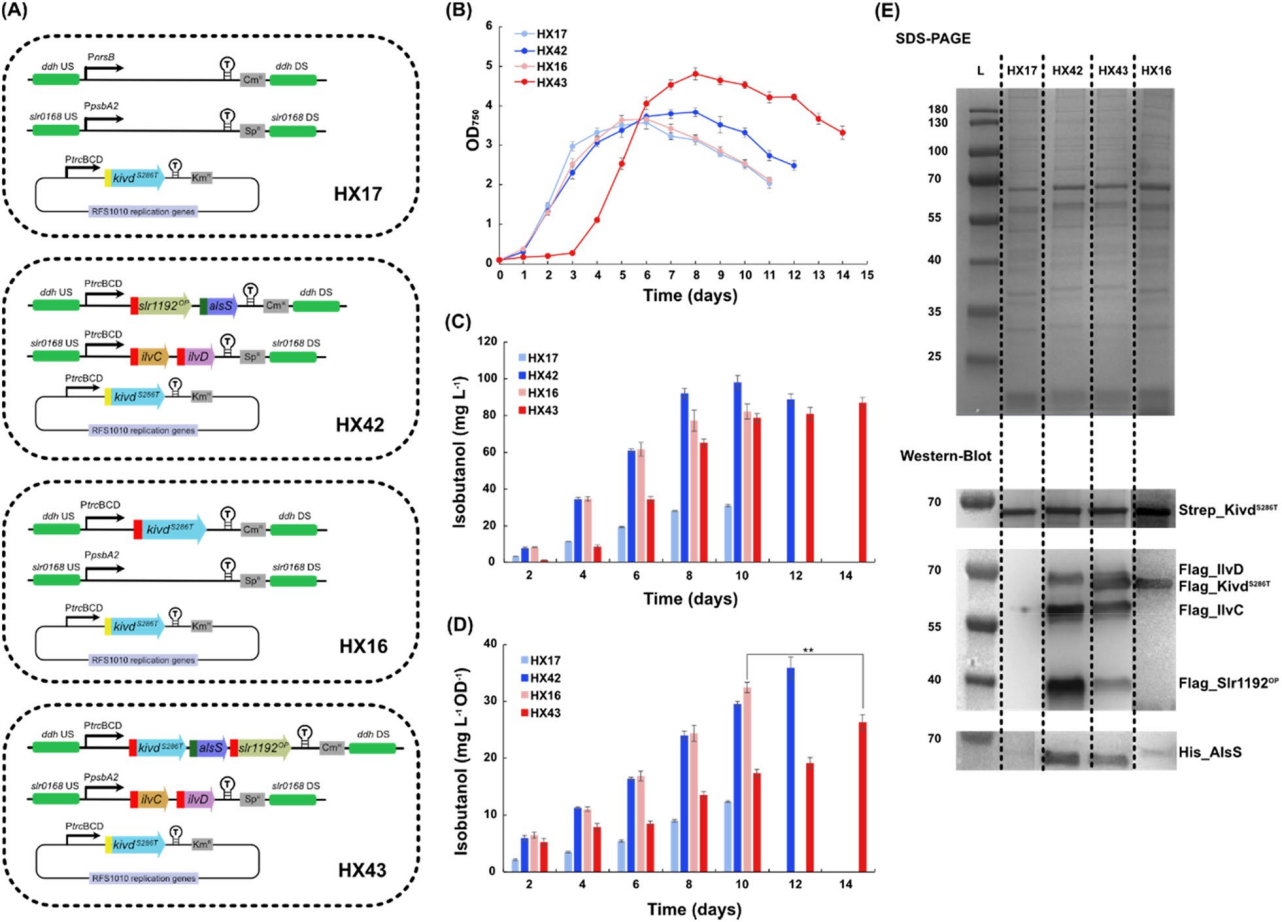
Schematic overview of genetic constructs used and comparison of growth, protein levels (SDS-PAGE/Western-immunoblot) and isobutanol production/isobutanol production per cell in engineered *Synechocystis* PCC 6803 strains HX17, HX42, HX16 and HX43. **A** Schematic presentation of the genetic constructs in the engineered strains. *kivd*^*S286T*^: encodes α-ketoisovalerate decarboxylase (*Lactococcus lactis*); *alsS*: encodes acetolactate synthase (*Bacillus subtilis*); *ilvC*: encodes acetohydroxy-acid isomeroreductase (*Escherichia coli*); *ilvD*: encodes dihydroxy-acid dehydratase (*E. coli*); *slr1192*^*OP*^: encodes codon optimized alcohol dehydrogenase (*Synechocystis*). HX16 and HX17 were generated as control strains. Kivd^S286T^ expressed on self-replicating plasmid was Strep-tagged at the N-terminal; IlvC, IlvD, Slr1192^OP^, and Kivd^S286T^ expressed in either the *ddh* (*slr1556*) site or the *slr0168* site of chromosome was Flag-tagged at the N-terminal. AlsS expressed in the *ddh* (*slr1556*) site of chromosome was His-tagged at the N-terminal. **B** Growth curves of the engineered strains during 14-day cultivation. **C** Isobutanol production of indicated strains. **D** Isobutanol production per cell of indicated strains. **E** SDS-PAGE (top) and Western-immunoblot (down). Each lane represents result from respective strain. L: ladder (in kDa). For SDS-PAGE, 5 μg of total soluble protein were loaded for each strain. For Western-immunoblot, 5 μg, 20 μg, and 20 μg of total soluble protein were loaded for each strain to detect Strep-tagged, Flag-tagged, and His-tagged protein, respectively. Results are the mean of three biological replicates, each with three technical replicates. Error bars represent standard deviation. Asterisk represents significant difference between different strains (one-way ANOVA, **p < 0.005)

As discussed above, similar isobutanol production per cell was observed in strains with Kivd^S286T^ solely expressed or Kivd^S286T^ expressed together with one or two selected enzymes from 2-keto acid pathway (Fig. 2C). Integrating *kivd* from *L. lactis* and *yqhD* from *E. coli* into the neutral site I (NSI) of the *Synechococcus* chromosome resulted in a production of 18 mg isobutanol L^−1^ [11]. After further integrating *alsS* from *B. subtilis* and *ilvC* and *ilvD* from *E. coli*, the final *Synechococcus* strain produced 450 mg isobutanol L^−1^, a 25-fold improvement [11]. Therefore, in order to further increase isobutanol production, integrating a complete 2-keto acid pathway into *Synechocystis* cell may be a promising strategy. Our further engineering of *Synechocystis* use AlsS from *B. subtilis*, IlvC and IlvD from *E. coli*, the engineered version of Kivd (Kivd^S286T^) [15], and the codon optimized version of Slr1192 (Slr1192^OP^) [23]. With five selected enzymes, two engineered strains with a complete 2-keto acid pathway integrated were generated, HX42 and HX43. In strain HX42, *slr1192*^*OP*^ and *alsS* were integrated into the *ddh* site and expression was driven by strong promoter P*trc*, *ilvC* and *ilvD* were integrated into the *slr0168* site and expression was driven by strong promoter P*trc*, and *Strep-kivd*^*S286T*^ was placed on self-replicating plasmid and expression was driven by strong promoter P*trc* (Fig. 3A). In strain HX43, *Flag-kivd*^*S286T*^, *alsS* and *slr1192*^*OP*^ were integrated into the *ddh* site and expression was driven by strong promoter P*trc*, *ilvC* and *ilvD* were integrated into the *slr0168* site and expression was driven by native promoter P*psbA2*, and *Strep-kivd*^*S286T*^ was placed on self-replicating plasmid and expression was driven by strong promoter P*trc* (Fig. 3A). Meanwhile, strains HX17 and HX16 were generated as control strains for HX42 and HX43, respectively (Fig. 3A). For HX17, chloramphenicol resistance (Cm^R^) cassette was integrated into the *ddh* site, spectinomycin resistance (Sp^R^) cassette was integrated into the *slr0168* site, and *Strep*-*kivd*^*S286T*^ placed on self-replicating plasmid. For HX16, *Flag*-*kivd*^*S286T*^ was integrated into the *ddh* site, spectinomycin resistance (Sp^R^) cassette was integrated into the *slr0168* site, and *Strep*-*kivd*^*S286T*^ placed on self-replicating plasmid.

Strain HX42 grew slightly slower than HX17 between days 2 and 5 (Fig. 3B). However, after day 6, the OD_750_ of HX17 started to decline while the OD_750_ of HX42 continued increasing until day 8 before declining from day 9 (Fig. 3B). Unexpectedly, HX43 had a much longer growth lag, with practically no cell growth observed during the first three days (Fig. 3B). However, thereafter HX43 started to grow and reached a highest density (OD_750_ = 4.8) on day 8 (Fig. 3B). Both strains HX42 and HX43 contain a complete 2-keto acid pathway, thus the distinct growth pattern of HX43 may due to the different expression units, e.g. promoters, and gene order within the operons (Fig. 3A).

**Fig. 4:**
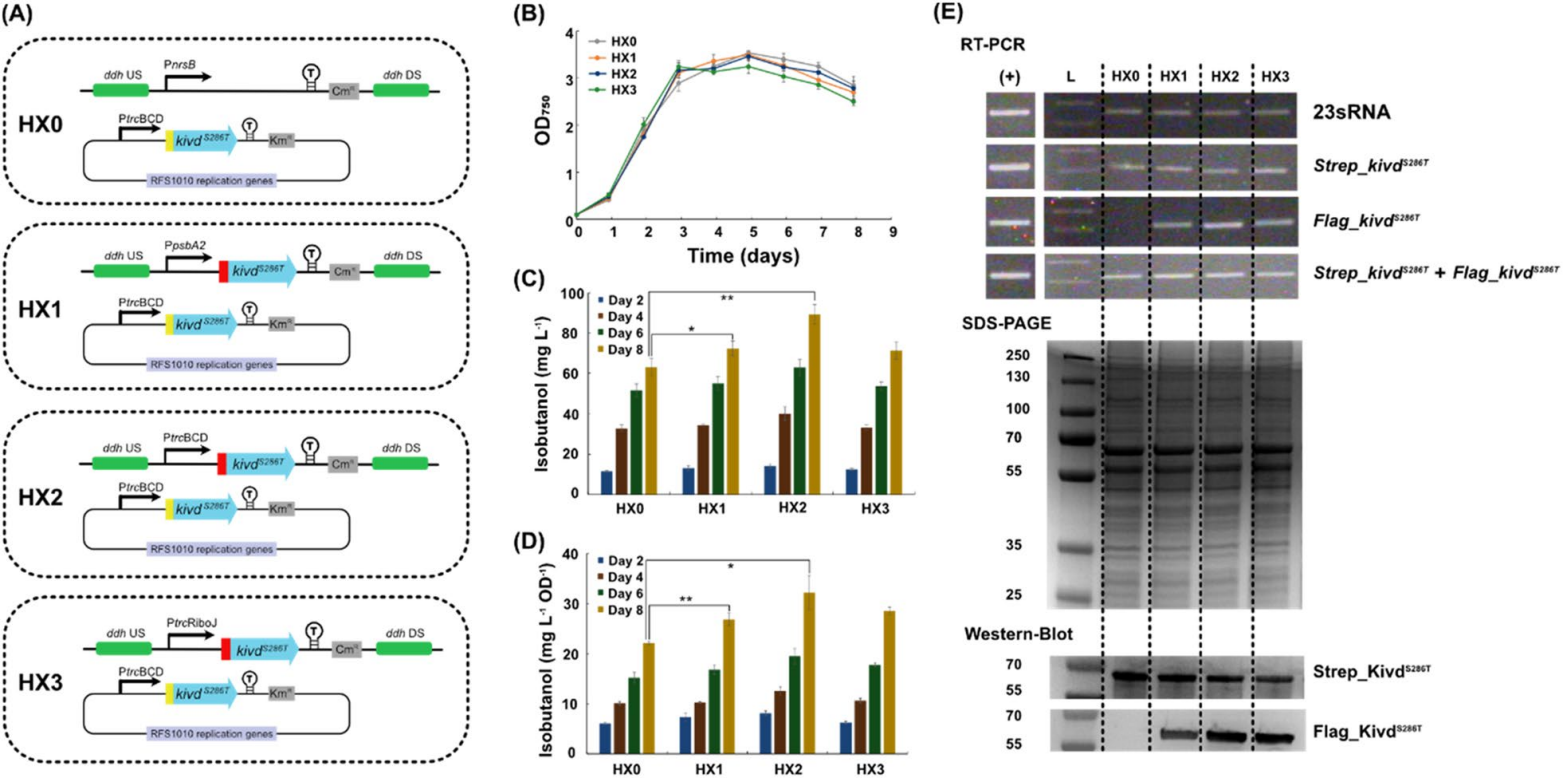
Schematic overview of genetic constructs used and comparison of growth, relative gene transcript (RT-PCR), protein levels (SDS-PAGE/Western-immunoblot) and isobutanol production/isobutanol production per cell in engineered *Synechocystis* PCC 6803 strains HX0, HX1, HX2, and HX3. **A** Schematic presentation of the genetic constructs in the engineered strains. *kivd*^*S286T*^: encodes α-ketoisovalerate decarboxylase (*Lactococcus lactis*). HX1, 2, 3 contain two copies of *kivd*^*S286T*^: first copy was put on a self-replicating plasmid and its expression was driven by P*trc*BCD; second copy was integrated in the *ddh* (*slr1556*) site of chromosome and its expression was driven by P*psbA2*, P*trc*BCD and P*trc*RiboJ, respectively. HX0 was generated as a control strain. Kivd^S286T^ expressed on self-replicating plasmids was Strep-tagged at the N-terminal; Kivd^S286T^ expressed in the *ddh* (*slr1556*) site of chromosome was Flag-tagged at the N-terminal. **B** Growth curves of the engineered strains during 8-day cultivation. **C** Isobutanol production at day 2, 4, 6, and 8 of indicated strains. **D** Isobutanol production per cell at day 2, 4, 6, and 8 of the indicated strains. **E** RT-PCR (top), SDS-PAGE (middle) and Western-immunoblot (bottom). Each lane represents result from respective strain. L: ladder (in kDa); (+): positive control, RT-PCR performed using *Synechocystis* genomic DNA or corresponding plasmid as template. For SDS-PAGE, 20 μg of total soluble protein were loaded for each strain. For Western-immunoblot, 5 μg and 20 μg of total soluble protein were loaded for each strain to detect Strep-tagged Kivd^S286T^ and Flag-tagged Kivd^S286T^, respectively. Results are the mean of three biological replicates, each with three technical replicates. Error bars represent standard deviation. Asterisk represents significant difference between different strains (one-way ANOVA, *p < 0.05, **p < 0.005)

The first engineered *Synechocystis* strain with a complete 2-keto acid pathway integrated, HX42, produced 98 mg isobutanol L^−1^ at day 10, while the control strain HX17 produced 31 mg L^−1^ (Fig. 3C). The expression of all five enzymes were confirmed by Western-immunoblot (Fig. 3E) with a 190% increase of isobutanol production per cell compared to the control strain HX17 (Fig. 3D). Interestingly, even though strains HX17 and HX0 contain the same *Strep-kivd*^*S286T*^ placed on self-replicating plasmid (Figs. 3A, 4A), the resulting isobutanol production of HX17 was only half of that of HX0 (Figs. 3C, 4C). A most likely reason is the different expression levels of Strep-Kivd^S286T^ (Fig. 2D, Fig. 3E; Additional file 1: Table S4). When compared to HX0, the difference of HX17 is that *slr0168* gene was replaced with an additional spectinomycin resistance (Sp^R^) cassette.

As Kivd^S286T^ has been identified as a bottleneck for isobutanol synthesis [12, 14], an additional copy, *Flag_kivd*^*S286T*^, was integrated into the *ddh* site in an operon together with *alsS* and *slr1192*^*OP*^, in order to increase the total expression of Kivd^S286T^ (strain HX43). HX43 produced 87 mg isobutanol L^−1^, which is similar to control strain HX16 (Fig. 3C). However, the isobutanol production per cell in HX43 was lower than in control strain HX16 (Fig. 3D), due to the higher optical density (OD_750_) of HX43 from day 6 until the end of cultivation (Fig. 3B). As shown in Western-immunoblot (Fig. 3E), the higher isobutanol production per cell of HX16 may be explained by the higher total expression level of Kivd^S286T^ (combined levels of Strep_Kivd^S286T^ and Flag_Kivd^S286T^) in HX16 compared to in HX43. Moreover, after integration of the *Flag_kivd*^*S286T*^ into the *ddh* site, the expression level of the other introduced genes in HX43 decreased significantly compared to in strain HX42 (Fig. 3E; Additional file 1: Table S4). As a consequence, the expression of the other enzymes of the 2-keto acid pathway may not have been enough to compensate for the decreased isobutanol production as a result from the decreased total Kivd^S286T^ expression level.

### Kivd^S286T^, an identified rate-limiting enzyme in 2-keto acid pathway for isobutanol synthesis

Our earlier study showed that engineered *Synechocystis* strain with *kivd*^*S286T*^ expressed on pEEK2, a self-replicating plasmid carrying the RSF1010 replicon, reached the highest isobutanol production [12]. As a key enzyme for isobutanol biosynthesis in *Synechocystis*, protein expression level of Kivd^S286T^ was demonstrated to be positively correlated with isobutanol production, and was regarded as the main bottleneck for further improvement of isobutanol production. Thus, in parallel with integration of a complete 2-keto acid pathway into *Synechocystis* cells, some efforts were invested to increase the expression level of Kivd^S286T^ in the cells with the aim of potentially enhancing isobutanol production.

Various approaches have been developed and applied to increase protein expression levels in *Synechocystis* cells [23–26]. Increasing gene dosage is an efficient approach to increase both gene transcription and translation, which has been successfully applied in *Synechocystis* for ethanol production [24], and in *Synechococcus* for squalene production [27]. 5′-region optimizations, including a selection of both promoters and genetic insulators, was systematically applied in *Synechocystis* resulting in significantly improved 1-butanol production [23].

Apart from placing one copy of *kivd*^*S286T*^ (*Strep_kivd*^*S286T*^) on pEEK2, a second copy (*Flag_kivd*^*S286T*^) was integrated into the *ddh* site of chromosome, to generate strain HX1 harboring two gene copies (Fig. 4A). The expression of *Flag_kivd*^*S286T*^ was driven by P*psbA2,* a native light intensity-dependent promoter [28]. HX0 was constructed as a control strain, with a single copy of *Strep_kivd*^*S286T*^ placed on pEEK2 and Cm^R^ cassette integrated into the *ddh* site (Fig. 4A). Similar growth patterns were observed for HX0 and HX1 (Fig. 4B), indicating gene dosage of *kivd*^*S286T*^ did not compromise cell growth. At day 8, isobutanol production of HX0 reached 63 mg L^−1^ (Fig. 4C) and strain HX1 was able to produce 72 mg isobutanol L^−1^ (Fig. 4C), which was 14% improvement compared to HX0. Similarly, a moderate increase of isobutanol production per cell was observed for HX1, up to 23% (Fig. 4D). This significant but still not dramatic increase may be explained by two factors: first, pEEK2 contains RSF1010 replicon which has a higher copy number (between 10 and 30) [29] than the *Synechocystis* chromosome (around 12) [30]; second, the promoter P*psbA2* is weaker in driving Kivd^S286T^ expression compared to P*trc* [14].

In strain HX1, P*psbA2* was used to drive *Flag-kivd*^*S286T*^ expression in the *ddh* site of chromosome. To attain higher Flag_Kivd^S286T^ protein expression for further isobutanol production improvement, P*psbA2* was replaced with the stronger promoter P*trc*, coupled with a BCD (bicistronic design) [31] or a RiboJ (self-cleaving ribozyme) [32], generating strains HX2 and HX3 (Fig. 4A). As expected, the translation level of *Flag-kivd*^*S286T*^ increased in HX2 and HX3, compared to HX1 (Fig. 4E; Additional file 1; Table S4). Both transcription and translation levels of *Flag_kivd*^*S286T*^ in HX2 was higher than in HX3 (Fig. 4E; Additional file 1: Table S4), indicating that the BCD element is more suitable to improve Flag-Kivd^S286T^ expression level. As the best performing strain, after 8-day cultivation, the isobutanol production and isobutanol production per cell of strain HX2 reached 89 mg L^−1^ and 32 mg L^−1^ OD^−1^, 42% and 46% increase respectively, compared to those achieved by HX0 (Fig. 4C, D). As for strain HX3, its isobutanol production and isobutanol production per cell reached similar level as HX1 (Fig. 4C, D), which may be explained by the similar total Kivd^S286T^ protein levels in both strains. Even though more Flag_Kivd^S286T^ expressed in HX3 than that expressed in HX1, less Strep_Kivd^S286T^ was expressed in HX3 than expressed in HX1, resulting in similar total Kivd^S286T^ protein expression level. The transcriptions of all *kivd*^*S286T*^ genes were confirmed using RT-PCR (Fig. 4E). The transcription level of *Strep_kivd*^*S286T*^ was similar in all four engineered strains, while strain HX2 showed the highest transcription level of *Flag_kivd*^*S286T*^ (Fig. 4E).

Both strategies of increasing gene dosage and replacement of a strong promoter resulted in improved isobutanol production in *Synechocystis*, which is consistent to what was observed for improved squalene production with the same strategies performed in *Synechococcus* [32]. After integrating a second copy of *Flag-kivd*^*S286T*^ in the *ddh* site, strains HX1, 2 and 3 showed higher isobutanol production than the control strain, which was positively related to total Kivd^S286T^ protein levels, as observed previously [12]. A decrease of Strep-Kivd^S286T^ protein expression was detected by Western-immunoblot in strains HX1, 2 and 3 (Fig. 4E; Additional file 1: Table S4). Two hypotheses could be made. First, when introducing a second copy of *Flag-kivd*^*S286T*^ in the chromosome, the copy numbers of the RSF1010 replicon-based plasmid declined, leading to less *Strep-kivd*^*S286T*^ gene dosage contributed from the self-replicating plasmid and resulting decreased Strep-Kivd^S286T^ expression. Second, *Flag*-*kivd*^*S286T*^ in the chromosome and *Strep-kivd*^*S286T*^ on self-replicating plasmid having identical gene sequences, encoding the same heterologous protein, may interfere with each other at transcription and translation level. This may lead to decreased protein expression level [33]. However, there was no significant difference of mRNA relative levels of *Strep-kivd*^*S286T*^ being observed between the four strains (Fig. 4E). This indicates similar mRNA levels of *Strep-kivd*^*S286T*^ on the self-replicating plasmid, supporting an interference on the translation level, but not on transcription, resulting in different protein expression levels.

Additional attempts were explored to improve Kivd^S286T^ expression levels. An effective approach improving β-phellandrene synthase protein expression has been demonstrated by fusing β-phellandrene synthase with phycocyanin β-subunit (encoded by *cpcB* gene), resulting in a 100-fold improvement of β-phellandrene hydrocarbons production in *Synechocystis* [25]. Therefore, to potentially improve Kivd^S286T^ expression level, different *kivd*^*S286T*^ fusion constructs were generated with the *Synechocystis* endogenous *cpcB* gene. However, no isobutanol production was detected (data not shown). Moreover, the small endogenous plasmid of *Synechocystis*, pCA2.4 is stable and has been shown as to have the potential to increase gene expression level [26]. However, only trace amount of isobutanol was detected after integrating *kivd*^*S286T*^ into a predicted neutral site of pCA2.4 (data not shown).

### Rewiring carbon flux by integrating third copy of ***kivd***^***S286T***^ in various sites of the chromosome

**Fig. 5:**
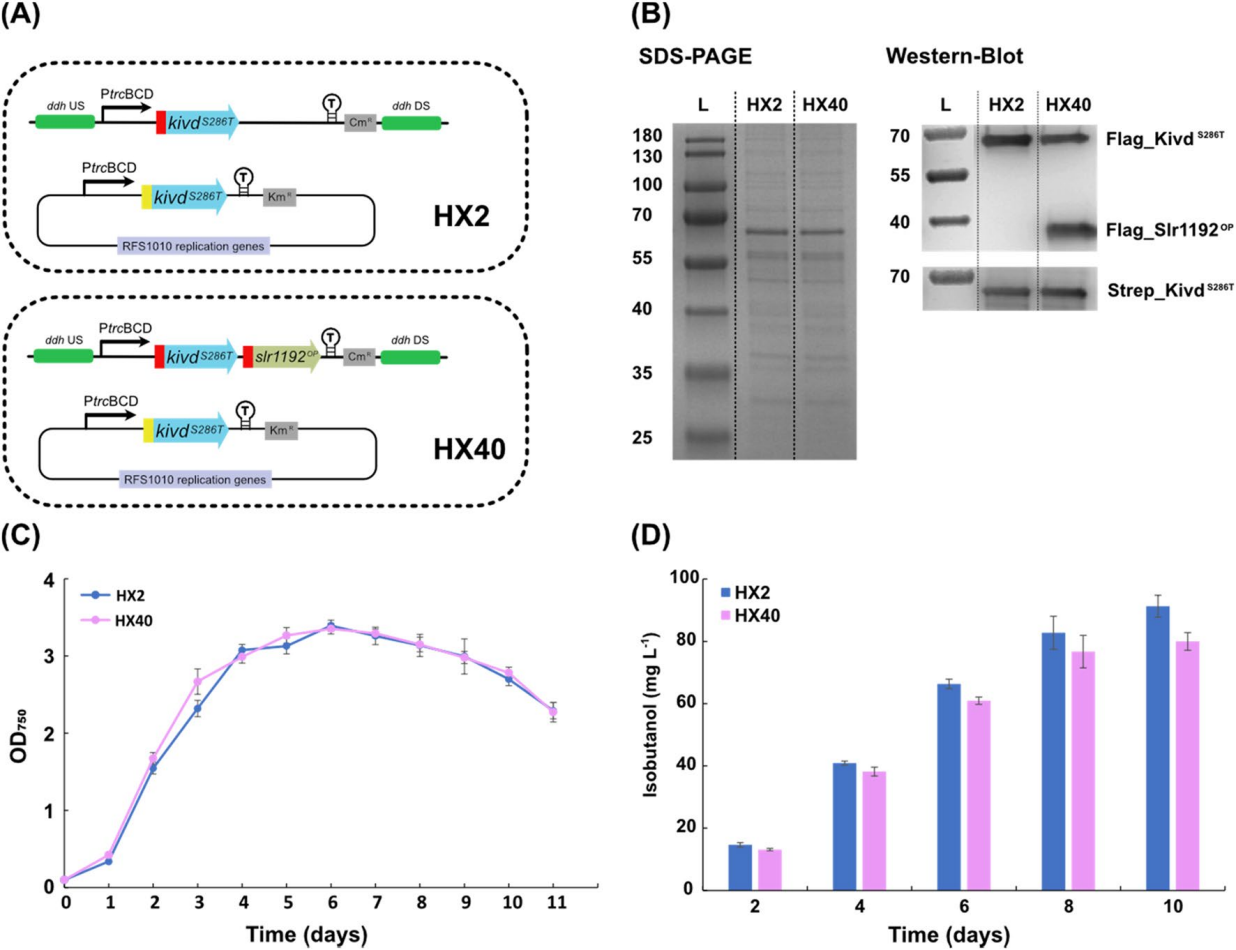
Schematic overview of genetic constructs used and comparison of growth, protein levels (SDS-PAGE/Western-immunoblot) and isobutanol production in engineered *Synechocystis* PCC 6803 strains HX2 and HX40. **A** Schematic presentation of the genetic constructs in the engineered strains. *kivd*^*S286T*^: encodes α-ketoisovalerate decarboxylase (*Lactococcus lactis*); *slr1192*^*OP*^: encodes codon optimized alcohol dehydrogenase (*Synechocystis*). Kivd^S286T^ expressed on self-replicating plasmids was Strep-tagged at the N-terminal; Slr1192^OP^ and Kivd^S286T^ expressed in the *ddh* (*slr1556*) site of chromosome was Flag-tagged at the N-terminal. **B** SDS-PAGE (left) and Western-immunoblot (right). Each lane represents result from respective strain. L: ladder (in kDa). For SDS-PAGE, 5 μg of total soluble protein were loaded for each strain. For Western-immunoblot, 5 μg and 20 μg of total soluble protein were loaded for each strain to detect Strep-tagged and Flag-tagged protein, respectively. **C** Growth curves of the engineered strains during 11-day cultivation. **D** Isobutanol production at day 2, 4, 6, and 8 of indicated strains. Results are the mean of three biological replicates, each with three technical replicates. Error bars represent standard deviation

After introducing a second copy of *Flag-kivd*^*S286T*^, the resulting strain HX2 was able to achieve higher isobutanol production, confirming Kivd^S286T^ as a key and rate-limiting enzyme in the 2-keto acid pathway for isobutanol synthesis. In strain HX2, it is unclear if Kivd^S286T^ is still the bottleneck, or the bottleneck is shifted to other enzymes in the isobutanol synthesis pathway (Fig. 1). Initially, in order to explore if Adh is the new bottleneck for isobutanol synthesis or not, strain HX40 was generated, with *Flag-kivd*^*S286T*^ and *slr1192*^*OP*^ co-expressed as an operon in the *ddh* site of chromosome (Fig. 5A). Similar growth patterns were observed for strains HX2 and HX40 (Fig. 5C), and protein expression was confirmed by Western-immunoblot (Fig. 5B). However, HX40 did not produced more isobutanol than strain HX2 (Fig. 5D), which with sole *Flag-kivd*^*S286*^ integrated in the *ddh* site of chromosome (Fig. 5A). Thus, the native alcohol dehydrogenases (encoded by *slr1192* and *slr0942*) are not bottlenecks for isobutanol production in strain HX2. As expected, when co-expressed with *slr1192*^*OP*^ in an operon in the chromosome, there was less Flag-Kivd^S286T^ protein expressed (strain HX40) when compared with expressed solely in the chromosome (strain HX2), which is consistent with what was observed earlier [14]. Therefore, at present stage, isobutanol production is still positively correlated with the total Kivd^S286T^ expression level, making Kivd^S286T^ a target for further investigations.

**Fig. 6:**
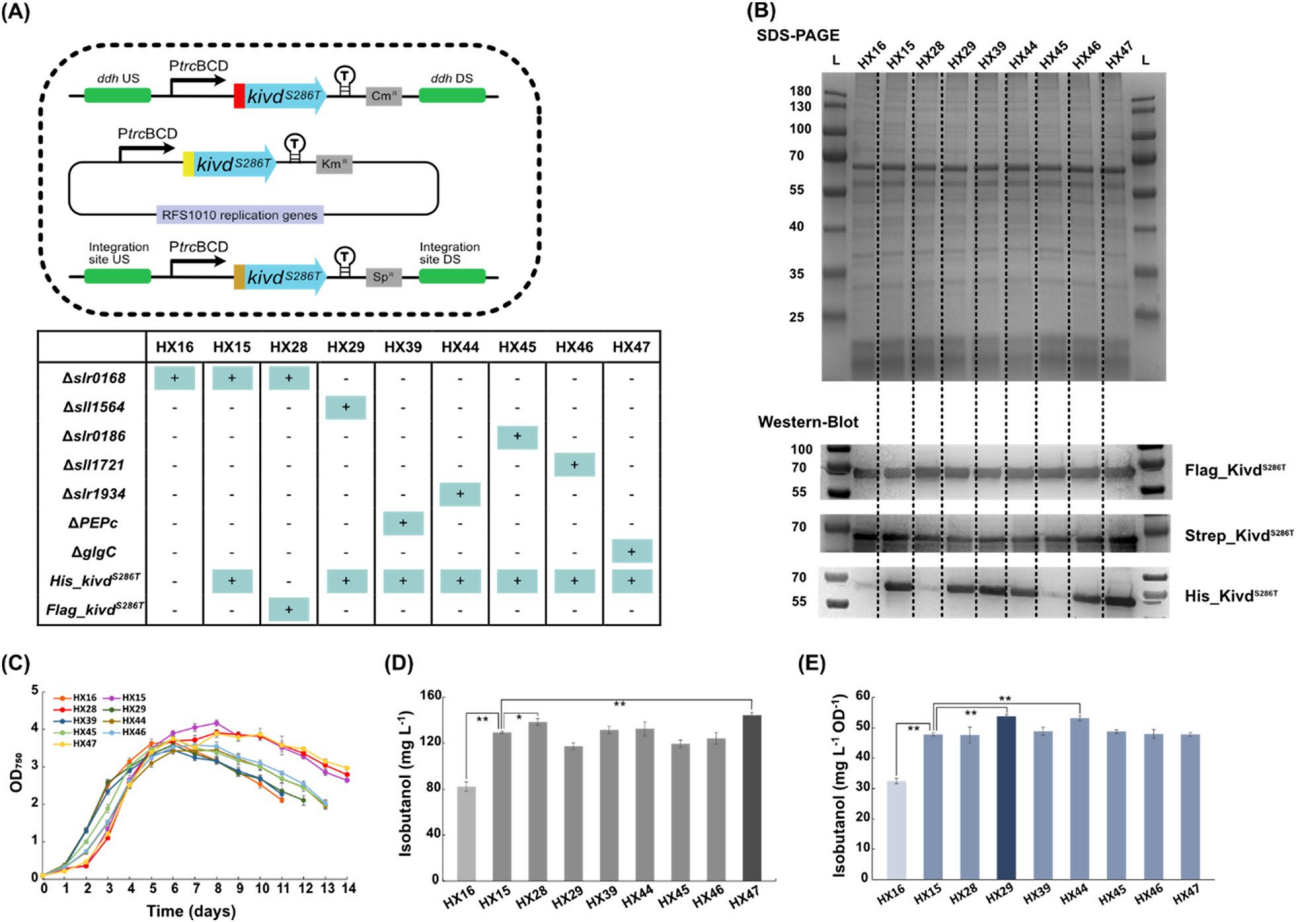
Schematic overview of genetic constructs used and comparison of growth, protein levels (SDS-PAGE/Western-immunoblot) and isobutanol production/isobutanol production per cell in engineered *Synechocystis* PCC 6803 strains HX15-16, HX28-29, HX39, and HX44-47. **A** Engineered *Synechocystis* strains with different integration sites for the third copy of *kivd*^*S286T*^. *slr0168*: encodes a hypothetical protein; *sll1564* and *slr0186*: encode 2-isopropylmalate synthase; *sll1721* and *slr1934*: encode pyruvate dehydrogenase; *PEPc*: encodes phosphoenolpyruvate carboxylase; *glgC*: encodes glucose-1-phosphate adenylyltransferase. HX16 was generated as a control strain. *kivd*^*S286T*^: encodes α-ketoisovalerate decarboxylase (*Lactococcus lactis*). Kivd^S286T^ expressed on self-replicating plasmids was Strep-tagged at the N-terminal; Kivd^S286T^ expressed in the *ddh* (*slr1556*) site of chromosome was Flag-tagged at the N-terminal; Kivd^S286T^ expressed in the third integration site of chromosome was either His-tagged or Flag-tagged at the N-terminal. **B** SDS-PAGE (top) and Western-immunoblot (bottom). Each lane represents result from respective strain. L: ladder (in kDa). For SDS-PAGE, 5 μg of total soluble protein were loaded for each strain. For Western-immunoblot, 5 μg, 20 μg, and 20 μg of total soluble protein were loaded for each strain to detect Strep-tagged, Flag-tagged, and His-tagged protein, respectively. **C** Growth curves of the engineered strains during 14-day cultivation. **D** Maximal isobutanol production observed of indicated strains. **E** Maximal isobutanol production per cell observed of indicated strains. “+” and “−” indicate with and without target gene manipulation, respectively. Results are the mean of three biological replicates, each with three technical replicates. Error bars represent standard deviation. Asterisk represents significant difference between different strains (one-way ANOVA, *p < 0.05, **p < 0.005)

Therefore, more efforts were made to further increase the total Kivd^S286T^ expression level in *Synechocystis* cells. In strain HX2, a strong P*trc*, coupled with BCD was used to drive the expression of *kivd*^*S286T*^ on self-replicating plasmid as well as in the *ddh* site of chromosome. It is challenging to further improve Kivd^S286T^ expression by tuning transcription and translation through systematically screening different promoters as well as ribosome binding sites (RBSs). Instead, further increasing gene dosage of *kivd*^*S286T*^ may be an efficient and straightforward approach to achieve a higher total Kivd^S286T^ expression level. The *ddh* site was used for the introduction of the second copy of *Flag-kivd*^*S286T*^ in the previous step, which may be a competing pathway for isobutanol synthesis. Initially, *slr0168*, encoding a hypothetical protein, was selected as an integration site for the third copy of *kivd*^*S286T*^, since it has been suggested *slr0168* knock-out has no effects on e.g. phenotypes and metabolisms in *Synechocystis* [34]. Two strains with three copies of *kivd*^*S286T*^ were generated: HX15 with a His-tagged *kivd*^*S286T*^ and HX28 with a Flag-tagged *kivd*^*S286T*^, both in the *slr0168* site (Fig. 6A). As control, strain HX16 with two copies of *kivd*^*S286T*^ was generated (Fig. 6A). Unexpectedly, both strains HX15 and HX28 showed a growth lag in the beginning of cultivation, but were able to achieve a higher maximum OD_750_ and survived for a longer period, until day 14, when compared with control strain HX16 (Fig. 6C). Maximal isobutanol production by HX15 and HX28 were 129 mg L^−1^ and 138 mg L^−1^, 57% and 68% improvements compared to by strain HX16 (Fig. 6D). It is interesting to observe the minor but distinct production differences between HX15 and HX28, maybe as a consequence of the different tags attached in the N terminal of Kivd^S286T^. Previously, protein engineering was successfully performed on Kivd and the strain introduced with the engineered Kivd^S286T^ showed more than threefold increase in the production of isobutanol [15]. By modulating the copy numbers of *kivd*^*S286T*^ in *Synechocystis* cells, the total Kivd^S286T^ protein expression increased, resulting a 2.7-fold increase of isobutanol production, compared to the previously best-performing isobutanol-producing strain pEEK2-ST [12].

The *slr0168* site was chosen as an integrate site for introduction of the third copy of *kivd*^*S286T*^. The resulting strains HX15 and HX28 both showed a lag phase. To alleviate growth deficiency caused by *slr0168* knock-out, it is important to further screen for other suitable integration sites to introduce the third copy of *kivd*^*S286T*^. As shown in Fig. 1, there are several competing pathways for isobutanol biosynthesis. Therefore, via integrating the third copy of *kivd*^*S286T*^ into different sites of chromosome, the overall cellular carbon flux may be modified to favor isobutanol synthesis. *glgC* encodes glucose-1-phosphate adenylyltransferase which catalyzes the first reaction of glycogen synthesis, a major carbohydrate storage compound in cyanobacteria. Δ*glgC* strain of *Synechococcus* showed significantly, 2.5 times, increased isobutanol production [13]. By integrating the third copy of *kivd*^*S286T*^ into the *glgC* site, strain HX47 was generated (Fig. 6A). The growth of HX47, similar to HX15, was impaired between days 0–2 when cultivated under 50 μmol photons m^−2^ s^−1^ (Fig. 6C), though it was previously reported that the growth retardation of Δ*glgC* mutant was only observed when cultivated under high light conditions (> 80 μmol photons m^−2^ s^−1^) [35]. As for isobutanol production, HX 47 produced 144 mg L^−1^ (Fig. 6D), slightly higher than strain HX15. Interestingly, HX47 continued to produce isobutanol, 4–6 mg L^−1^ OD^−1^ d^−1^, for 8 days, resulting in the highest production in short-term screening experiments so far (Table 2). In contrast to what has been reported before in *Synechococcus* [13], no dramatic improvement in isobutanol production was observed in Δ*glgC Synechocystis strain* HX47. This may be explained by the experimental conditions used including different light intensities.

**Table 2:** Isobutanol production rate per cell in the engineered *Synechocystis* PCC 6803 strains

Strain (mg L^−1^ OD^−1^ day^−1^)	Time					
	Day 0–1	Day 2–3	Day 4–5	Day 6–7	Day 8–9	Day 10–11
**HX16**	3.2 ± 0.26	**4.2 ± 0.20**	3.7 ± 0.32	2.4 ± 0.59	1.0 ± 0.38	
**HX15**	4.2 ± 0.21	3.9 ± 0.31	**4.7 ± 0.02**	4.6 ± 0.29	2.7 ± 0.52	1.7 ± 0.22
**HX28**	4.1 ± 0.22	4.5 ± 0.06	**5.3 ± 0.13**	4.1 ± 0.27	3.4 ± 0.22	2.1 ± 0.18
**HX29**	5.4 ± 0.19	**6.0 ± 0.29**	4.6 ± 0.27	3.9 ± 0.11	1.7 ± 0.36	
**HX39**	6.2 ± 0.26	**6.6 ± 0.13**	5.9 ± 0.19	3.9 ± 0.42	2.2 ± 0.24	
**HX44**	**6.0 ± 0.17**	**6.0 ± 0.25**	5.5 ± 0.20	4.9 ± 0.10	3.7 ± + 0.66	
**HX45**	**6.1 ± 0.45**	5.3 ± 0.20	4.9 ± 0.24	3.5 ± 0.15	2.5 ± 0.17	
**HX46**	5.1 ± 0.55	**5.3 ± 0.24**	5.2 ± 0.39	4.8 ± 0.37	2.8 ± 0.35	
**HX47**	5.3 ± 0.32	4.8 ± 0.20	**5.4 ± 0.16**	4.2 ± 0.20	4.7 ± 0.30	0.9 ± 0.17

The highest isobutanol production rate per cell of each strain is shown in bold. Results are the mean of three biological replicates, each with three technical replicates. Errors represent standard deviation

Pyruvate is the precursor of the 2-keto acid pathway, a central metabolite which is further metabolized into different essential metabolites in various metabolic pathways. Thus, increasing the pyruvate pool is a relevant strategy to provide more precursor for isobutanol synthesis. In *Synechocystis*, pyruvate is generated from phosphoenolpyruvate (PEP), catalyzed by pyruvate kinase (PK), while phosphoenolpyruvate carboxylase (PEPc) is competing with PK for the shared substrate PEP (Fig. 1). Enhanced 2-ketoisovalerate production was observed after knocking out *PEPc* in the bacterium *Corynebacterium glutamicum* [36]. Therefore, *PEPc* (*sll0920*) was chosen as an integration site to introduce the third copy of *kivd*^*S286T*^ to generate strain HX39 (Fig. 6A). As suspected, we were unable to generate a fully segregated strain, since PEPc is a key enzyme for carbon fixation in cyanobacteria with a main function to produce oxaloacetate (OAA) to feed into the Tricarboxylic acid (TCA) cycle (Fig. 1). Intermediates of the TCA cycle are used for synthesis of glutamate and aspartate family amino acids. Because of the same reason, instead of knocking out/knocking down *PEPc* in *Synechococcus*, Cheah et al. [37] heterologously introduced a revert reaction catalyzed by the enzyme PEP carboxykinase (PCK) from *E. coli* to convert OAA into PEP. Strain HX39 grew as fast as control strain HX16 between days 0 and 4, reaching slightly lower OD_750_ than HX16 (Fig. 6C). After 10-day cultivation, the final isobutanol production was 132 mg L^−1^ (Fig. 6D), similar to in HX15. Interestingly, among all the engineered strains, strain HX39 showed the highest production rate per cell (6.6 mg L^−1^ OD^−1^ d^−1^) between days 2 and 3 (Table 2).

Apart from increasing PEP availability for pyruvate synthesis, another approach to increase pyruvate pool is to decrease the enzymatic reactions using pyruvate as substrate. One important enzymatic reaction using pyruvate as substrate is catalyzed by pyruvate dehydrogenase complex for acetyl-CoA synthesis (Fig. 1). In *Synechocystis,* the pyruvate dehydrogenase complex consists of three enzymes: pyruvate dehydrogenase (encoded by *sll1721* and *slr1934*), dihydrolipoamide acetyltransferase (encoded by *sll1841*), and dihydrolipoamide dehydrogenase (encoded by *slr1096*). Since pyruvate dehydrogenase is regarded as a rate-limiting step for this reaction, both *sll1721* and *slr1934* were assigned as integration sites for the introduction of the third copy of *kivd*^*S286T*^ to generate strains HX46 and HX44 (Fig. 6A). We were unable to get fully segregated cells for both strains, as pyruvate dehydrogenase complex catalyzes the main route for acetyl-CoA synthesis, which is further involved in TCA cycle and used for e.g. lipid synthesis. Even though the growth rates of HX44 and HX46 between days 0 and 4 were slower than control strain HX16 and faster than strain HX15 (Fig. 6C), both strains produced similar isobutanol as HX15, 132 mg L^−1^ and 124 mg L^−1^, respectively (Fig. 6D). However, after normalizing isobutanol production to per cell, strain HX44 reached 53 mg L^−1^ OD^−1^, which is significantly higher than control strain HX15 (Fig. 6E).

As presented previously, engineered *Synechocystis* cells produced two end-products, isobutanol and 3M1B, after heterologous expression of *kivd* from *L. lactis* [14]. Based on that, protein engineering was performed on Kivd, resulting in improved isobutanol production as well as an improved isobutanol-to-3M1B molar ratio [15]. To further shift the carbon flow from 3M1B to isobutanol synthesis, *leuA*, encoding 2-isopropylmalate synthase, was selected as a target for the third copy of *kivd*^*S286T*^ integration (Fig. 1). Limited information is available for 2-isopropylmalate synthase of *Synechocystis*, however, two genes, *slr0186* and *sll1564*, are annotated as *leuA*. Thus, two strains were generated, HX45 and HX29, through the integration of the third copy of *kivd*^*S286T*^ in the *slr0186* site and *sll1564* site (Fig. 6A), respectively. Interestingly, strain HX29 got fully segregated, but not strain HX45. Furthermore, a higher isobutanol-to-3M1B molar ratio was observed for strain HX45, when compared to strain HX15 (Additional file 1: Fig. S3). Both facts indicate that *slr0186* encoding LeuA plays a major role in leucine synthesis as well as 3M1B synthesis. For *sll1564* encoding LeuA, apart from LeuA activity, it is also annotated as citramalate synthase. Thus, complete knock-out of *slr0186* is lethal for *Synechocystis cells*. Consistently, HX29 grew as fast as control strain HX16, while HX45 showed slower growth between days 0 and 4. After 12-day cultivation, isobutanol production per cell of HX45 reached 49 mg L^−1^ OD^−1^ (Fig. 6E), and HX29 reached 54 mg L^−1^ OD^−1^, the highest isobutanol production per cell of all engineered strains (Fig. 6E).

Multiple integration sites have been experimentally examined for effects on isobutanol production. By knock-out/knock-down of the discussed genes encoding key enzymes of potential competing pathways, more carbon flux is expected to be directed towards isobutanol synthesis. The highest isobutanol production, 144 mg L^−1^, was obtained by strain HX47 (Fig. 6D), 76% improvement compared to control strain HX16. Strain HX29 showed the highest isobutanol production per cell, 54 mg L^−1^ OD^−1^ (Fig. 6E), 69% improvement compared to control strain HX16.

Some strains were not fully segregated, meaning instead of knocked out, the selected competing pathway was knocked down. When comparing the His-tagged Kivd^S286T^ expression levels of all engineered strains, similar expression level was observed between strains fully segregated and strain not fully segregated (Fig. 6B). Interestingly, HX47, a strain not fully segregated even had more His-tagged Kivd^S286T^ expressed than other strains (Fig. 6B; Additional file 1: Table S4). From these results, it may be inferred that in strains without fully segregation, the majority of the chromosomes already contain *kivd*^*S286T*^ in the assigned integration sites. Furthermore, when a protein-coding gene *kivd*^*S286T*^ was introduced into different integration sites of the chromosome, the resulting protein expression levels were not consistent (Fig. 6B), a result from different genomic context with regulations in both transcription and translation levels [38]. On the other hand, when knocking out essential genes with a traditional homologous double recombination method, it is sometimes difficult to get fully segregated strains. Alternatively, antisense RNA [37] may be employed as a tool for downregulation of those essential genes to further optimize isobutanol production in *Synechocystis*.

Compared to strain HX15, the newly generated engineered strain HX47 showed significantly increased isobutanol production whereas strains HX29 and HX44 showed significantly increased isobutanol production per cell (Fig. 6D, E). Three hypotheses can be made: first, there is no obvious effect when knocking out/knocking down selected competing pathways singly, providing a new investigation direction to knock out/knock down all of them simultaneously to effectively concentrate carbon flow into the 2-keto acid pathway for isobutanol synthesis. As an approach for simultaneous repression of multiple genes, clustered regularly interspaced short palindromic repeats interference (CRISPRi) has been developed for *Synechocystis* [39], and was successfully used to improve fatty alcohol production in *Synechocystis* [40]; Second, knock-out/knock-down of competing pathways is effective for enhancement of precursor availability for isobutanol synthesis, however, Kivd^S286T^ is still a bottleneck for isobutanol synthesis in *Synechocystis* cells. Thus, to resolve the bottleneck Kivd^S286T^, two approaches may be adopted: one is to further improve its catalytic activity by protein engineering based on structure analysis as well as computational simulation; another one is to develop new strategies for further improvement of Kivd^S286T^ protein amount in *Synechocystis* cells; Third, after introducing three copies of *kivd*^*S286T*^ in *Synechocystis* cells, enough Kivd^S286T^ are ready to convert 2-ketoisovalerate into isobutyraldehyde. Therefore, Kivd^S286T^ is not the rate-limiting enzyme for isobutanol synthesis. Instead, in this stage, there is (are) other bottleneck(s) in isobutanol synthesis pathway, and further investigation are needed to precisely find out the potential bottleneck(s). Regarding the last two hypotheses, only after successfully removing bottleneck(s), the contribution of competing pathway knock-out/knock-down could be manifested from the resulting isobutanol production in engineered *Synechocystis* cells.

## Conclusion

**Fig. 7:**
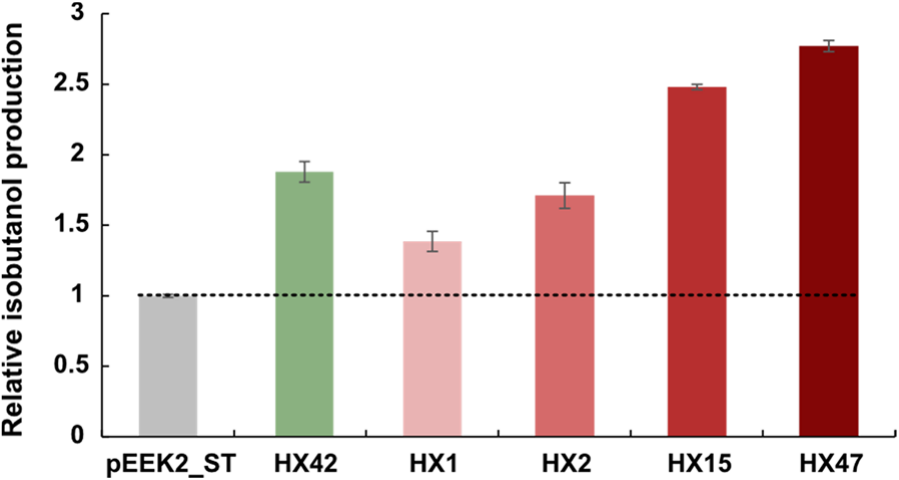
Relative maximal isobutanol production (mg L^−1^) of engineered *Synechocystis* PCC 6803 strains HX1, 2, 15, 42 and 47 in short-term screening experiments, compared to the previously best isobutanol-producing strain *Synechocystis* PCC 6803 pEEK2_ST [12]. Results are the mean of three biological replicates, each with three technical replicates. Error bars represent standard deviation. For detailed strain information, see Table 1

In summary, this is the first demonstration of the feasibility to generate an engineered *Synechocystis* strain with a complete 2-keto acid pathway integrated. In short-term screening experiments, the resulting strain HX42 was able to produce 98 mg isobutanol L^−1^, an 88% increase compared to the previously reported best-producing strain pEEK2-ST (Fig. 7) [12]. By fine-tuning expression of the key enzyme Kivd^S286T^ from both transcription and translation levels, the resulting strain HX2 and HX15 showed 70% and 148% increase of isobutanol production compared with strain pEEK2-ST (Fig. 7). Further improved isobutanol production, 144 mg L^−1^, was obtained by interrupting the glycogen biosynthesis pathway (strain HX47) (Fig. 7). The present results suggest that a combination of integrating a complete 2-keto acid pathway and meanwhile keeping high protein expression of Kivd^S286T^ is a potential strategy to further improve isobutanol production in *Synechocystis*. Moreover, further simultaneous repression of multiple competing pathways of isobutanol synthesis pathway is likely to concentrate more carbon flux into the 2-keto acid pathway for isobutanol synthesis. The final strain HX47 was able to reach much higher isobutanol production and production rate if cultivated in a condition with precisely controlled pH, as detailed before [12]. Finally, in order to successfully use *Synechocystis* as a novel platform for production of photosynthetic isobutanol, one key factor is to develop a chemostat system suitable for cyanobacteria cultivation, combined with an automatic isobutanol harvesting and upgrade system.

## Supplementary Information

**Additional file 1. Fig. S1**: Schematic overview of genetic constructs used and Western-immunoblot results of engineered *Synechocystis* PCC 6803 strains HX0 and HX6. (**A**) Schematic presentation of the genetic constructs in the engineered strains. *kivd*^*S286T*^: encodes α-ketoisovalerate decarboxylase (*Lactococcus lactis*). *alsS*: encodes acetolactate synthase (*Bacillus subtilis*). *Kivd*^*S286T*^ expressed on self-replicating vectors was Strep-tagged at the N-terminal; AlsS expressed in the *ddh* (*slr1556*) site of chromosome was His-tagged at the N-terminal. (**B**) Western-immunoblot results of strains HX0 and HX6. Each lane represents result from respective strain. 5 μg and 20 μg of total soluble protein were loaded for each strain to detect Strep-tagged Kivd^S286T^ and His-tagged AlsS, respectively. **Fig. S2**: Comparison of growth in engineered *Synechocystis* PCC 6803 strains HX0, HX5, HX7, HX8, and HX9 during 8-day cultivation. Results are the mean of three biological replicates, each with three technical replicates. Error bars represent standard deviation. **Fig. S3**: Schematic overview of genetic constructs used and comparison of molar ratio of isobutanol and 3-methyl-1-butanol (3M1B) of engineered *Synechocystis* PCC 6803 strains HX15, HX29, and HX45. (**A**) Molar ratio of isobutanol and 3M1B of indicated strains, calculated based on the isobutanol production measured on day 4. (**B**) Schematic presentation of the genetic constructs in the engineered strains. Asterisk represents significant difference between HX45 and HX15 (One-way ANOVA, p < 0.05). Results are the mean of three biological replicates, each with three technical replicates. Error bars represent standard deviation. **Table S1**: Sequences of codon optimized synthetic genes used in this study. **Table S2**: Plasmids used in this study. Expressed genes in bold. **Table S3**: Oligonucleotides used in this study. **Table S4**: Expression quantification of heterologously expressed enzymes. The expression level of each protein is presented by the corresponding band intensity. The unit is intensity x mm

## Data Availability

The datasets used during the current study are available from the corresponding author on reasonable request.

## Abbreviations

3M1B: 3-Methyl-1-butanol
Adh: Alcohol dehydrogenase
AlsS: Acetolactate synthase
BCD: Bicistronic design
Cm^R^: Chloramphenicol resistance
CRISPRi: Clustered regularly interspaced short palindromic repeats interference
IlvC: Acetohydroxy-acid isomeroreductase
IlvD: Dihydroxy-acid dehydrase
Kivd: α-Ketoisovalerate decarboxylase
NSI: Neutral site I
OAA: Oxaloacetate
OD_750_: Optical density
PCK: PEP carboxykinase
PEP: Phosphoenolpyruvate
PEPc: Phosphoenolpyruvate carboxylase
PK: Pyruvate kinase
RBSs: Ribosome binding sites
RiboJ: Self-cleaving ribozyme
Rubisco: Ribulose 1,5-bisphosphate carboxylase/oxygenase
Slr1192^OP^: Codon optimized version of Slr1192
Sp^R^: Spectinomycin resistance
*Synechococcus*: *Synechococcus elongatus* PCC 7942
*Synechocystis*: *Synechocystis* PCC 6803
TCA cycle: Tricarboxylic acid cycle
