# Supplementary material for: Expressing 2-keto acid pathway enzymes significantly increases photosynthetic isobutanol production
Source: Microb Cell Fact. 2022 Feb 1;21:17. doi: 10.1186/s12934-022-01738-z (PMC8805274; doi:10.1186/s12934-022-01738-z)
Supplement: Supplementary file 1 — Additional file 1. Fig. S1: Schematic overview of genetic constructs used and Western-immunoblot results of engineered Synechocystis PCC 6803 strains HX0 and HX6. (A) Schematic presentation of the genetic constructs in the engineered strains. kivdS286T: encodes α-ketoisovalerate decarboxylase (Lactococcus lactis). alsS: encodes acetolactate synthase (Bacillus subtilis). KivdS286T expressed on self-replicating vectors was Strep-tagged at the N-terminal; AlsS expressed in the ddh (slr1556) site of chromosome was His-tagged at the N-terminal. (B) Western-immunoblot results of strains HX0 and HX6. Each lane represents result from respective strain. 5 μg and 20 μg of total soluble protein were loaded for each strain to detect Strep-tagged KivdS286T and His-tagged AlsS, respectively. Fig. S2: Comparison of growth in engineered Synechocystis PCC 6803 strains HX0, HX5, HX7, HX8, and HX9 during 8-day cultivation. Results are the mean of three biological replicates, each with three technical replicates. Error bars represent standard deviation. Fig. S3: Schematic overview of genetic constructs used and comparison of molar ratio of isobutanol and 3-methyl-1-butanol (3M1B) of engineered Synechocystis PCC 6803 strains HX15, HX29, and HX45. (A) Molar ratio of isobutanol and 3M1B of indicated strains, calculated based on the isobutanol production measured on day 4. (B) Schematic presentation of the genetic constructs in the engineered strains. Asterisk represents significant difference between HX45 and HX15 (One-way ANOVA, p < 0.05). Results are the mean of three biological replicates, each with three technical replicates. Error bars represent standard deviation. Table S1: Sequences of codon optimized synthetic genes used in this study. Table S2: Plasmids used in this study. Expressed genes in bold. Table S3: Oligonucleotides used in this study. Table S4: Expression quantification of heterologously expressed enzymes. The expression level of each protein is presented by the correspo [file 12934_2022_1738_MOESM1_ESM.pdf]

Expressing 2-keto acid pathway enzymes significantly increases photosynthetic isobutanol production

Hao Xie, Peter Lindblad*

hao.xie@kemi.uu.se

peter.lindblad@kemi.uu.se

Microbial Chemistry, Department of Chemistry-Ångström Laboratory, Uppsala University, Box 523, SE-75120 Uppsala, Sweden

Additional File 1

Figs. S1 – S3

Tables S1 – S4

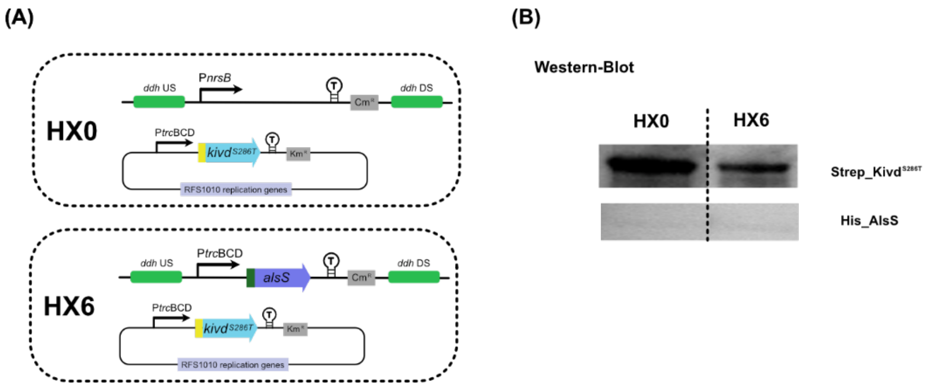

Fig. S1: Schematic overview of genetic constructs used and Western-immunoblot results of engineered *Synechocystis* PCC 6803 strains HX0 and HX6. (A) Schematic presentation of the genetic constructs in the engineered strains. *kivd*^{S286T}: encodes α-ketoisovalerate decarboxylase (*Lactococcus lactis*). *alsS*: encodes acetolactate synthase (*Bacillus subtilis*). Kivd^{S286T} expressed on self-replicating vectors was Strep-tagged at the N-terminal; AlsS expressed in the *ddh* (*slr1556*) site of chromosome was His-tagged at the N-terminal. (B) Western-immunoblot results of strains HX0 and HX6. Each lane represents result from respective strain. 5 µg and 20 µg of total soluble protein were loaded for each strain to detect Strep-tagged Kivd^{S286T} and His-tagged AlsS, respectively.

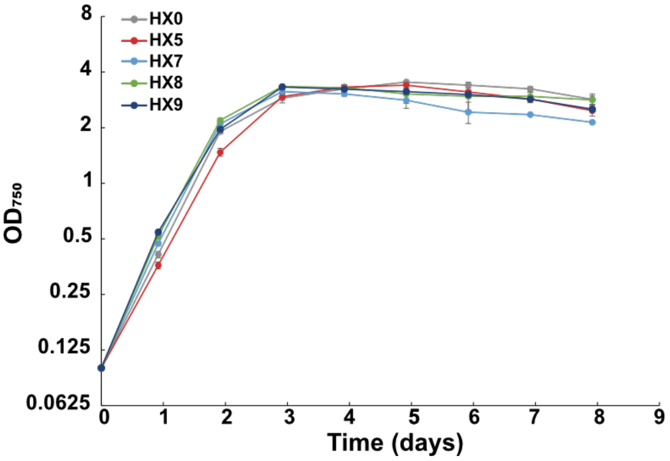

Fig. S2: Comparison of growth in engineered *Synechocystis* PCC 6803 strains HX0, HX5, HX7, HX8, and HX9 during 8-day cultivation. Results are the mean of three biological replicates, each with three technical replicates. Error bars represent standard deviation.

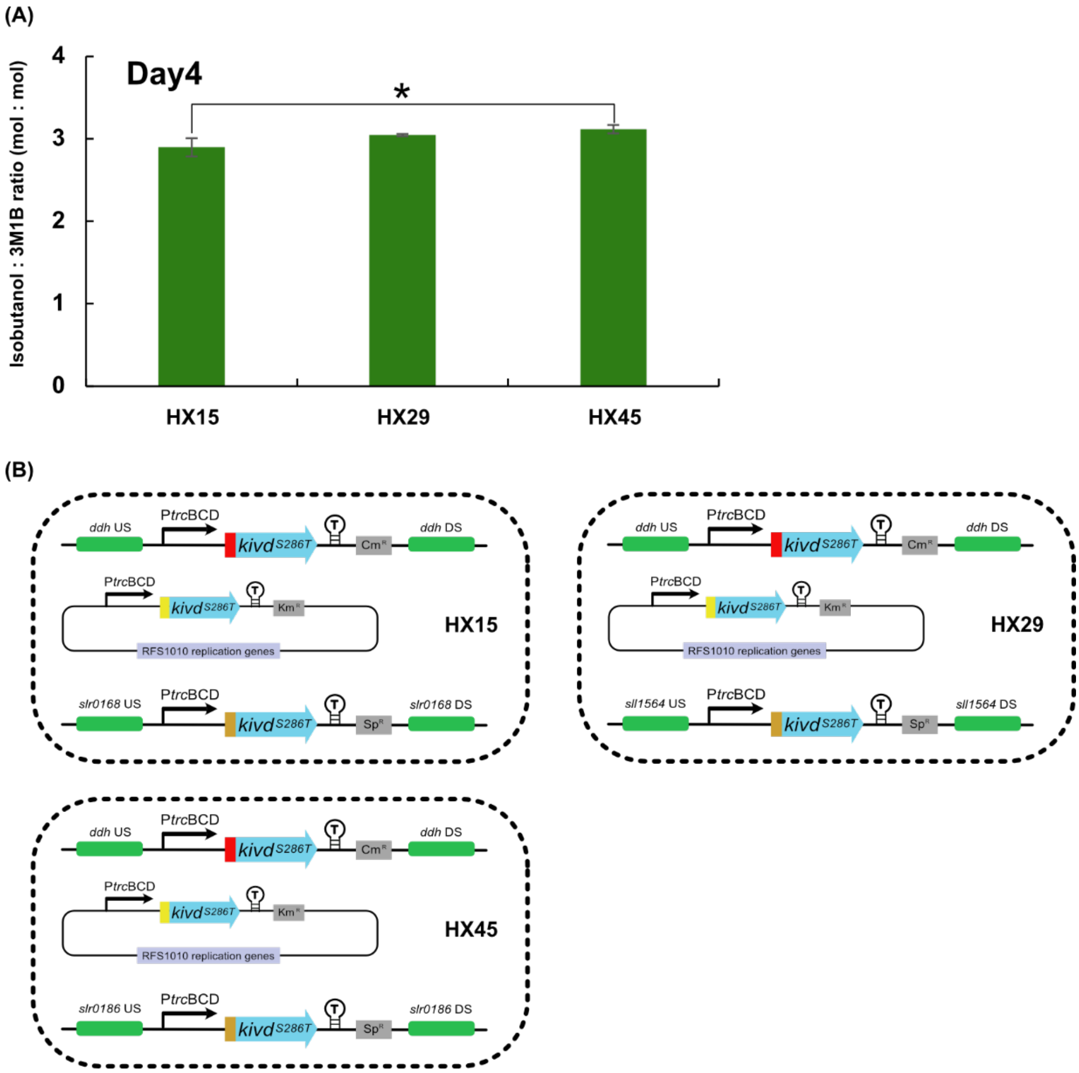

Fig. S3: Schematic overview of genetic constructs used and comparison of molar ratio of isobutanol and 3-methyl-1-butanol (3M1B) of engineered *Synechocystis* PCC 6803 strains HX15, HX29, and HX45. (A) Molar ratio of isobutanol and 3M1B of indicated strains, calculated based on the isobutanol production measured on day 4. (B) Schematic presentation of the genetic constructs in the engineered strains. Asterisk represents significant difference between HX45 and HX15 (One-way ANOVA, $p < 0.05$). Results are the mean of three biological replicates, each with three technical replicates. Error bars represent standard deviation.

Table S1. Sequences of codon optimized synthetic genes used in this study.

Gene	Nucleotide sequence (5'-3')
kivd^{S286T}	ATGTACACCGTGGGAGATTACTTACTGGACCGCTTACATGAACTGGGTATTGAA<br>GAAATTTTTGGCGTGCCCGGAGATTACAACTTACAATTTTTAGATCAAATTATTA<br>GTCATAAAGACATGAAATGGGTGGGTAATGCCAACGAACTGAATGCCTCCTATA<br>TGGCTGATGGGTACGCCCGTACCAAAAAAGCCGCTGCCTTTTTGACCACTTTTG<br>GCGTGGGTGAACTGTCCGCCGTTAATGGCTTGGCTGGTAGTTATGCCGAAAAC<br>TTACCCGTGGTTGAAATTGTGGGGTCCCCCACCTCCAAAGTTCAAAACGAAGGA<br>AAATTTGTGCATCACACCTTGGCCGATGGCGACTTTAAACATTTTATGAAAATGC<br>ACGAACCCGTGACTGCTGCCCGGACCTTGTTAACTGCCGAAAATGCTACCGTG<br>GAAATTGATCGTGTTCTGAGTGCTCTGTTGAAAGAACGGAAACCCGTGTACATT<br>AACTTGCCCGTGGACGTTGCTGCCGCTAAAGCCGAAAAACCCTCCTTACCCCT<br>GAAAAAAGAAAATTCCACCAGTAACACTTCTGATCAAGAAATTTTAAACAAAATT<br>CAGGAATCCCTGAAAAACGCCAAAAAACCCATTGTTATTACTGGCCACGAAATT<br>ATTAGTTTTGGTTTGGAAAAAACCGTGACTCAATTTATTTCCAAAACCAAATTGC<br>CCATTACCACTCTGAATTTTGGGAAATCCAGTGTGGATGAAGCCCTGCCCTCCT<br>TTTTGGGCATTTATAATGGCACCTTGAGTGAACCCAACCTGAAAGAATTTGTTGA<br>AAGCGCTGATTTTATTTTGATGCTGGGGGTGAAATTGACCGACACCTCTACTGG<br>AGCCTTTACCCATCACCTGAACGAAAACAAAATGATTAGTCTGAACATTGATGAA<br>GGTAAAATCTTTAACGAACGGATTCAAAACTTTGATTTTGAATCCTTGATTTCCA<br>GTTTACTGGACTTATCTGAAATTGAATACAAAGGGAAATACATTGATAAAAAACA<br>GGAAGACTTTGTGCCCAGCAATGCTTTGTTATCTCAAGATCGCCTGTGGCAGGC<br>CGTTGAAAATTTGACTCAAAGTAACGAAACCATTGTGGCTGAACAGGGCACCTC<br>CTTTTTCGGAGCCAGCTCTATTTTTCTGAAAAGCAAATCTCATTTTATTGGACAA<br>CCCTTATGGGGGAGTATTGGATATACCTTTCCCGCCGCTTTGGGCAGCCAGATT<br>GCCGATAAAGAATCTCGCCACCTGTTGTTTATTGGGGACGGCTCCTTGCAATTG<br>ACCGTTCAGGAATTAGGCCTGGCCATTCGCGAAAAAATTAACCCCATTTGTTTTA<br>TTATTAACAACGATGGCTACACCGTGGAACGTGAAATTCATGGTCCCAATCAAT<br>CCTATAACGACATTCCCATGTGGAATTACTCCAAATTGCCCGAAAGTTTTGGTGC<br>CACTGAAGATCGGGTGGTTTCTAAAATTGTGCGCACCGAAAATGAATTTGTGAG<br>CGTTATGAAAGAAGCCCAGGCTGACCCCAACCGTATGTACTGGATTGAATTGAT<br>TCTGGCCAAAGAAGGTGCCCCCAAAGTGCTGAAGAAAATGGGAAAACTGTTTG<br>CTGAACAGAATAAAAGCTAA
alsS	ATGTTGACCAAAGCCACTAAAGAACAAAAATCCTTAGTGAAAAACCGCGGTGCC<br>GAATTGGTGGTTGATTGTTTAGTGGAACAGGGGGTGACCCATGTTTTTGGGATT<br>CCCGGAGCCAAAATTGATGCTGTGTTTGACGCCTTGCAAGATAAAGGTCCCGAA<br>ATTATTGTGGCCCGCCACGAACAAAACGCTGCTTTTATGGCCCAGGCTGTGGG<br>GCGTTTAACCGGCAAACCCGGTGTGGTTCTGGTTACTAGCGGCCCCGGTGCTT<br>CTAATTTAGCCACCGGGTTGTTAACCGCCAACACTGAAGGAGATCCCGTGGTTG<br>CCCTGGCTGGCAATGTGATTCGGGCCGACCGCTTGAAACGTACCCATCAATCC<br>CTGGATAACGCCGCTTTGTTTCAGCCCATTACTAAATATAGTGTGGAAGTTCAAG<br>ATGTGAAAAATATTCCCGAAGCTGTTACCAACGCCTTTCGTATTGCCTCCGCTG<br>GGCAAGCCGGAGCCGCTTTTGTGAGTTTTCCCCAGGATGTGGTTAACGAAGTTA<br>CCAACACTAAAAACGTGCGGGCCGTTGCCGCTCCCAAATTGGGACCCGCCGCT<br>GATGACGCCATTAGCGCCGCTATTGCTAAAATTCAAACCGCCAAATTGCCCGTG<br>GTTCTGGTGGGGATGAAAGGCGGTCGCCCCGAAGCCATTAAAGCTGTGCGTAA<br>ACTGTTGAAAAAAGTGCAATTACCCTTTGTTGAAACCTATCAGGCCGCTGGTACT<br>TTGAGTCGGGATTTAGAAGACCAATACTTTGGCCGTATTGGTTTATTTCGGAATC<br>AACCCGGAGACTTACTGTTGGAACAGGCCGATGTGGTTCTGACCATTGGCTATG

	ACCCCATTGAATACGATCCCAAATTTTGGAACATTAACGGCGACCGCACTATTAT<br>TCATTTGGATGAAATTATTGCTGATATTGACCACGCCTATCAACCCGACTTAGAA<br>CTGATTGGTGATATTCCCAGTACCATTAATCATATTGAACACGATGCTGTGAAAG<br>TTGAATTTGCCGAACGTGAACAGAAAATTCTGAGCGATTTGAAACAATACATGCA<br>TGAAGGGGAACAGGTGCCCGCTGACTGGAAATCTGATCGGGCCCACCCCTTGG<br>AAATTGTGAAAGAATTACGCAATGCCGTTGATGACCATGTGACCGTTACTTGCG<br>ATATTGGCTCCCACGCCATTTGGATGTCCCGGTATTTTCGCAGTTACGAACCCT<br>TGACCTTAATGATTAGCAACGGCATGCAAACTTTAGGCGTGGCTTTGCCCTGGG<br>CTATTGGAGCTTCTTTAGTGAAACCCGGTGAAAAAGTGGTTTCCGTTAGTGGTG<br>ATGGGGGATTTCTGTTTAGTGCTATGGAATTGGAAACCGCTGTGCGGTTAAAAG<br>CCCCCATTGTGCATATTGTTTGGAATGACTCCACCTATGATATGGTGGCCTTTCA<br>ACAGTTGAAAAAATACAATCGCACCTCCGCCGTGGATTTTGGCAACATTGATATT<br>GTTAAATACGCTGAATCCTTTGGGGCCACCGGATTACGTGTGGAATCTCCCGAC<br>CAATTAGCTGATGTTCTGCGGCAGGGGATGAATGCCGAAGGACCCGTGATTATT<br>GATGTGCCCGTTGATTACAGCGACAATATTAACTTAGCCTCTGATAAACTGCCC<br>AAAGAATTTGGCGAACTGATGAAAACCAAAGCCTTGTAA
ilvC	ATGGCCAACTATTTTAACACCCTGAACTTACGCCAACAACTGGCTCAACTGGGG<br>AAATGCCGCTTTATGGGCCGTGACGAATTTGCTGACGGAGCCAGTTATTTGCAA<br>GGCAAAAAAGTGGTTATTGTGGGCTGTGGTGCCCAAGGGCTGAATCAGGGATT<br>GAACATGCGCGATTCCGGTCTGGACATTAGTTATGCCTTGCGTAAAGAAGCTAT<br>TGCCGAAAAACGTGCCTCCTGGCGGAAAGCTACCGAAAACGGCTTTAAAGTGG<br>GTACTTACGAAGAATTAATTCCCCAAGCCGATTTAGTTATTAATCTGACCCCCGA<br>TAAACAACATAGCGACGTGGTTCGGACTGTGCAGCCCTTAATGAAAGATGGGG<br>CCGCTCTGGGATATTCTCACGGCTTTAATATTGTGGAAGTTGGGGAACAAATTC<br>GCAAAGACATTACCGTGGTTATGGTGGCCCCCAAATGCCCCGGCACTGAAGTT<br>CGCGAAGAATACAAACGTGGGTTTGGAGTGCCCACCTTGATTGCCGTTCATCCC<br>GAAAATGATCCCAAAGGCGAGGGTATGGCTATTGCCAAAGCCTGGGCCGCTGC<br>CACCGGCGGTCACCGTGCTGGCGTGCTGGAATCCAGTTTTGTGGCTGAAGTTA<br>AATCCGATTTGATGGGTGAACAAACCATTTTGTGTGGCATGTTACAGGCCGGGA<br>GTTTGCTGTGCTTTGATAAATTAGTGGAAGAAGGAACCGACCCCGCCTATGCTG<br>AAAAATTGATTCAATTTGGGTGGGAAACCATTACTGAAGCCCTGAAACAAGGCG<br>GCATTACCTTAATGATGGATCGTCTGTCCAACCCCGCCAAACTGCGGGCCTACG<br>CTCTGAGTGAACAATTGAAAGAAATTATGGCCCCCTTATTTCAGAAACACATGGA<br>TGACATTATTTCCGGAGAATTTAGCTCTGGCATGATGGCCGATTGGGCTAATGA<br>TGACAAAAAACTGTTGACCTGGCGCGAAGAAACCGGCAAAACTGCCTTTGAAAC<br>TGCTCCCCAATATGAAGGGAAAATTGGAGAACAGGAATACTTTGATAAGGGTGT<br>GTTGATGATTGCTATGGTGAAAGCTGGGGTTGAATTAGCCTTTGAAACTATGGT<br>GGATAGTGGTATTATTGAAGAAAGCGCCTATTACGAATCTTTGCATGAATTGCCC<br>TTAATTGCCAATACCATTGCTCGGAAACGCTTATATGAAATGAACGTGGTTATTA<br>GCGATACTGCCGAATATGGCAATTACTTGTTTTCTTACGCTTGTGTGCCCTTACT<br>GAAACCCTTTATGGCCGAATTGCAACCCGGCGACTTAGGCAAAGCCATTCCCG<br>AAGGTGCTGTGGATAATGGGCAGTTACGGGACGTTAACGAAGCCATTCGCTCC<br>CACGCTATTGAACAGGTTGGGAAAAAATTGCGTGGATACATGACCGATATGAAA<br>CGGATTGCTGTGGCTGGTTAA
ilvD	ATGCCCAAATATCGTAGTGCCACCACTACCCACGGCCGTAATATGGCTGGTGC<br>CCGCGCTTTGTGGCGTGCTACTGGAATGACCGACGCTGATTTTGGTAAACCCAT<br>TATTGCCGTGGTTAACTCCTTTACCCAATTTGTGCCCGGCCATGTGCACCTGCG<br>GGATTTGGGAAAATTAGTGGCCGAACAGATTGAAGCCGCTGGCGGTGTTGCTA<br>AAGAATTTAATACCATTGCCGTGGATGACGGGATTGCTATGGGACATGGGGGAA<br>TGCTGTATAGTTTGCCCAGCCGTGAATTAATTGCCGATTCTGTTGAATACATGGT<br>GAATGCTCACTGCGCCGACGCTATGGTGTGCATTAGCAACTGTGATAAAATTAC<br>TCCCGGCATGTTGATGGCCTCTTTGCGCTTAAATATTCCCGTTATTTTTGTGTCC<br>GGCGGTCCTATGGAAGCCGGTAAAACCAAATTGAGTGACCAAATTATTAAACTG<br>GACTTGGTGGATGCCATGATTCAGGGGGCTGATCCCAAAGTTTCCGACAGTCA<br>AAGCGATCAGGTGGAACGCAGTGCCTGTCCCACCTGCGGCTCTTGTTCCGGCA<br>TGTTTACTGCCAATAGCATGAACTGTTTGACCGAAGCCTTGGGGCTGTCTCAAC<br>CCGGTAATGGCTCCTTGTTAGCCACTCATGCTGATCGCAAACAGTTGTTTTTAAA

	CGCCGGCAAACGCATTGTGGAATTGACCAAACGTTACTACGAACAAAACGATGA<br>AAGTGCCTTACCCCGTAACATTGCTAGCAAAGCCGCTTTTGAAAATGCCATGAC<br>CCTGGATATTGCTATGGGCGGCTCCACCAACACCGTGTTGCACCTGTTGGCCG<br>CTGCCCAAGAAGCCGAAATTGATTTTACTATGATGAGTGACATTGATAAACTGAG<br>CCGTAAAGTTCCCCAATTGTGTAAAGTGGCCCCCTCCACCCAGAAATATCACAT<br>GGAAGATGTTCACCGGGCCGGCGGTGTGATTGGGATTTTAGGAGAACTGGACC<br>GGGCCGGGTTACTGAATCGCGATGTTAAAAACGTGCTGGGCTTGACTTTACCC<br>CAAACCTTAGAACAGTACGATGTTATGCTGACCCAAGATGACGCCGTGAAAAAT<br>ATGTTTCGTGCTGGACCCGCTGGTATTCGCACTACCCAAGCCTTTAGTCAGGAC<br>TGCCGGTGGGATACCTTAGATGACGATCGTGCCAACGGCTGTATTCGGTCTCT<br>GGAACATGCTTATTCCAAAGATGGGGGATTGGCCGTGTTATACGGCAATTTTGC<br>TGAAAACGGTTGCATTGTTAAAACTGCCGGCGTGGACGATTCTATTTTGAAATTT<br>ACCGGTCCCGCCAAAGTTTATGAATCCCAAGACGATGCCGTGGAAGCTATTTTA<br>GGCGGTAAAGTGGTTGCCGGTGATGTGGTTGTGATTCGGTATGAAGGGCCCAA<br>AGGGGGACCCGGAATGCAAGAAATGTTGTACCCCACCTCTTTTCTGAAAAGTAT<br>GGGCTTGGGTAAAGCCTGTGCTCTGATTACTGATGGACGTTTTTCCGGCGGCA<br>CCTCCGGATTATCCATTGGACACGTGAGCCCCGAAGCTGCCAGTGGGGGAAGC<br>ATTGGGTTAATTGAAGACGGCGATTTGATTGCCATTGACATTCCCAATCGGGGC<br>ATTCAATTGCAGGTGTCCGATGCCGAATTAGCTGCTCGTCGGGAAGCTCAAGAT<br>GCTCGCGGTGATAAAGCCTGGACTCCCAAAAATCGTGAACGGCAAGTTAGTTTT<br>GCCTTGCGCGCCTACGCTAGTTTAGCCACCAGCGCTGACAAAGGTGCCGTGCG<br>TGATAAATCCAAATTAGGCGGTTAA
slr1192^{OP}	ATGATTAAAGCCTACGCCGCCTTGGAAGCCAATGGAAAATTACAGCCCTTTGAA<br>TACGATCCCGGTGCCCTGGGTGCTAACGAAGTTGAAATTGAAGTGCAATATTGT<br>GGCGTGTGTCATAGTGATTTGTCCATGATTAACAACGAATGGGGTATTAGTAAC<br>TATCCCTTAGTGCCCGGTCATGAAGTGGTGGGGACCGTGGCTGCTATGGGCGA<br>AGGTGTGAATCATGTGGAAGTGGGGGATTTAGTGGGGTTGGGCTGGCATAGTG<br>GCTATTGTATGACCTGTCATAGTTGTTTGTCCGGTTATCATAACTTGTGTGCCAC<br>CGCCGAATCCACCATTGTGGGGCATTATGGCGGTTTTGGCGATCGGGTGCGCG<br>CCAAAGGGGTGAGTGTGGTGAAATTGCCCAAAGGCATTGATTTGGCCTCCGCC<br>GGTCCCTTATTTTGTGGTGGCATTACCGTGTTTAGTCCTATGGTGGAATTGTCCT<br>TAAAACCCACCGCCAAAGTGGCCGTGATTGGCATTGGTGGGTTAGGTCATTTG<br>GCCGTGCAATTTTTGCGTGCCTGGGGCTGTGAAGTGACCGCCTTTACCAGTTC<br>CGCCCGGAAACAAACCGAAGTGTTGGAATTAGGGGCCCATCATATTTTAGATAG<br>TACCAATCCCGAAGCCATTGCCTCCGCCGAAGGCAAATTTGATTACATTATTAGT<br>ACCGTGAACTTGAAATTGGATTGGAACTTGTACATTTCCACCTTGGCTCCCCAA<br>GGTCATTTTCATTTTGTGGGCGTGGTGTTGGAACCCTTGGATTTAAATTTGTTTC<br>CCTTGTTGATGGGTCAACGCTCCGTGAGTGCTTCCCCCGTGGGGAGTCCCGCC<br>ACCATTGCCACCATGTTAGATTTTGCCGTGCGTCATGATATTAAACCCGTGGTG<br>GAACAATTTTCCTTTGATCAAATTAACGAAGCCATTGCTCATTTGGAAAGTGGTA<br>AAGCCCATTATCGGGTGGTGCTGTCCCATTCTAAAAACTAA

Table S2. Plasmids used in this study. Expressed genes in bold.

Plasmid	Relevant characteristics[a]	Reference
pEEK2-ST	pEEK2-(P*trc*BCD-***kivd***^{S286T}-T)-Km^R	(Miao et al., 2017)
pHX1	pDdh-(P*nrsB*-T)-Cm^R	This study
pHX2	pDdh-(P*psbA2*-***alsS***-T)-Cm^R	This study
pHX3	pDdh-(P*trc*BCD-***alsS***-T)-Cm^R	This study
pHX4	pDdh-(P*trc*BCD-***kivd***^{S286T}-***alsS***-T)-Cm^R	This study
pHX5	pDdh-(P*trc*BCD-***ilvD***-***alsS***-T)-Cm^R	This study
pHX6	pDdh-(P*trc*BCD-***slr1192***^{OP}-***alsS***-T)-Cm^R	This study
pHX7	pDdh-(P*psbA2*-***kivd***^{S286T}-T)-Cm^R	This study
pHX8	pDdh-(P*trc*BCD-***kivd***^{S286T}-T)-Cm^R	This study
pHX9	pDdh-(P*trc*RiboJ-***kivd***^{S286T}-T)-Cm^R	This study
pHX10	pDdh-(P*trc*BCD-***kivd***^{S286T}-***slr1192***^{OP}-T)-Cm^R	This study
pHX11	pDdh-(P*trc*BCD-***kivd***^{S286T}-***alsS***-***slr1192***^{OP}-T)-Cm^R	This study
pHX12	pNSI-(P*psbA2*-T)-Sp^R	This study
pHX13	pNSI-(P*trc*BCD-***ilvC***-***ilvD***-T)-Sp^R	This study
pHX14	pNSI-(P*psbA2*-***ilvC***-***ilvD***-T)-Sp^R	This study
pHX15	pNSI-(P*trc*BCD-***kivd***^{S286T}-T)-Sp^R	This study
pHX16	pSll1564-(P*trc*BCD-***kivd***^{S286T}-T)-Sp^R	This study
pHX17	pSlr0186-(P*trc*BCD-***kivd***^{S286T}-T)-Sp^R	This study
pHX18	pSll1721-(P*trc*BCD-***kivd***^{S286T}-T)-Sp^R	This study
pHX19	pSll1934-(P*trc*BCD-***kivd***^{S286T}-T)-Sp^R	This study
pHX20	pPEPc-(P*trc*BCD-***kivd***^{S286T}-T)-Sp^R	This study
pHX21	pGlgC-(P*trc*BCD-***kivd***^{S286T}-T)-Sp^R	This study

[a] NSI, neutral site I (*slr0168*); Km^R, Kanamycin resistance cassette; Sp^R, spectinomycin resistance cassette; Cm^R: chloramphenicol resistance cassette; T, Terminator BBa_B0015

Table S3. Oligonucleotides used in this study.

Primer name	Oligonucleotides sequence
**A. Primers for PCR amplification of homologous recombination regions**	
ddh_US_BglII_F	TATAAGATCTAAACCACTGGGCCAGTAGTTC
ddh_US_Ter_EcoRI_R	TATAGAATTCAAAAAAAGGATCTCAAGAAGATCCTTTGATTTTGACGATTATGGGAAGTAGTTTAG
ddh_DS_BamHI_F	TATAGGATCCGGTTAGAAAATATCAATGTTAAC
ddh_DS_SalI_R	TATAGTCGACCGGACTATTTGGTAGAACATAAAATC
slr0168_US_BglII_F	TATAAGATCTAATGTGGAACGGGGCCTAGACAC
slr0168_US_Ter_EcoRI_R	TATAGAATTCAAAAAAAGGATCTCAAGAAGATCCTTTGATTTTAGATTAATTCAACAGTAATATTTC
slr0168_DS_BamHI_F	TATAGGATCCCTCAGGGGCATTATCGGAGCAAG
slr0168_DS_XhoI_R	TATACTCGAGGATCGCCAAAGATGTTGGCCGTCGGG
sll1564_US_BglII_F	TATAAGATCTACAATTGATGATTAATCACCCAG
sll1564_US_Ter_EcoRI_R	TATAGAATTCAAAAAAAGGATCTCAAGAAGATCCTTTGATTTTAATTCAAGCGACGTTCAAGT
sll1564_DS_BamHI_F	TATAGGATCCTATCTGCTTGTTAGAATTTTAAAA
sll1564_DS_SalI_R	TATAGTCGACTGGTTAAGTTTGAGCATAAAACC
slr0186_US_BglII_F	TATAAGATCTTCGTCCAGGGTGGCTGGCAA
slr0186_US_Ter_EcoRI_R	TATAGAATTCAAAAAAAGGATCTCAAGAAGATCCTTTGATTTTAATAAACGCACCTTTTCAAGGG
slr0186_DS_BamHI_F	TATAGGATCCTGGCCCGGTGACAGTTACG
slr0186_DS_SalI_R	TATAGTCGACAGCTGGCATCGATCGCCTGTTGTA
sll1721_US_BglII_F	TATAAGATCTATCCCCACGAATATTAAGGGTG
sll1721_US_Ter_EcoRI_R	TATAGAATTCAAAAAAAGGATCTCAAGAAGATCCTTTGATTTTGATTTCTGGGGGAAAACTTAGT
sll1721_DS_BamHI_F	TATAGGATCCTTGGCCTCAAAACAGTACC
sll1721_DS_SalI_R	TATAGTCGACAGATTCCATTGGACAACTTTTAG
slr1934_US_BglII_F	TATAAGATCTATGCCCTGCCAGAGCCAATC
slr1934_US_Ter_EcoRI_R	TATAGAATTCAAAAAAAGGATCTCAAGAAGATCCTTTGATTTTAGTACAATTTGCAGTAGATAAAG
slr1934_DS_BamHI_F	TATAGGATCCTATGCTGGGGGAGCGGCG
slr1934_DS_SalI_R	TATAGTCGACCCAGTACGGATAGAGCAGCA
sll0920_US_BglII_F	TATAAGATCTATTGCCCTTCCACTGCATCC
sll0920_US_Ter_EcoRI_R	TATAGAATTCAAAAAAAGGATCTCAAGAAGATCCTTTGATTTTGGGAACCTCTAGGACAAGATG
sll0920_DS_BamHI_F	TATAGGATCCTCCAGTGATATGGTGCCTAAT

sll0920_DS_SalI_R	TATAGTCGACACGCCAATCTCCAGGGGGCCA
slr1176_US_BglII_F	TATAAGATCTAATTTTGGGGTGATTCCTTCC
slr1176_US_Ter_EcoRI_R	TATAGAATTCAAAAAAAGGATCTCAAGAAGATCCTTTGATTTTTTCGAAGTCAAGTTTAGAACCG
slr1176_DS_BamHI_F	TATAGGATCCGGCCAGTTTCTTTCCTCGCAC
slr1176_DS_SalI_R	TATAGTCGACATACTGGTCAACACATTTACG
**B. Primers for *Synechocystis* colony PCR to verify genomic integration**	
ddh_UUS_F	CAGCCGTACCACTTCTTCTAC
slr0168_UUS_F	ATGTGGAGTTTGTGGGTATTG
sll1564_UUS_F	AACAAACCGTTACCCCGGAC
sll0920_UUS_F	GTTTCCCCGGGCAAAATAGCTG
slr1934_UUS_F	GTTAAGGGCGGTCTAGTTTG
slr0186_UUS_F	CGTCCTCCATTTCTGCCCAC
sll1721_UUS_F	GCCATGGCAGTTTTCACTTC
slr1176_UUS_F	ACTCCAGGTAATCTTCTTTGG
CmR_SR	CTGAAATGCCTCAAAATGTTCTTTACG
kivd^{S286T}_SR	TTGGCATTACCCACCCATTTC
alsS_SR	CGCTAGTAACCAGAACCACAC
ilvD_SR	ATCTGTTCGGCCACTAATTTTC
slr1192^{OP}_SR	AGTTACTAATACCCCATTCGTTG
SpR_SR	GAGTCGATACTTCGGCGATC
ilvC_SR	CGCATGTTCAATCCCTGATTC
**C. Primers for PCR with *Synechocystis* genomic DNA to examine fully segregation**	
ddh_SF	AGTCCATGTACCATCCTATTCTC
ddh_SR	GCTCTTCCTCTTCTTCGTAAAC
slr0168_SF	CGGCAATGATCCAGAGAATG
slr0168_SR	CTATCTGCCAAAGCTGCTTC
sll1564_SF	GCTGGAGGGATTACAAACTAG
sll1564_SR	AGTACTAGTGAGATGGGCTAG
sll0920_SF	TGCAAGCTTCTGTAGGTACG
sll0920_SR	TTACCGAAGGGTTGCCGTCC
slr1934_SF	GTAATGGCGGAACTGTTTGG
slr1934_SR	GGATCCGCTAGGGAGTGACC
slr0186_SF	AACACCGTATTCACACCTTC

slr0186_SR	GGGCGACCTAAAAAGGGATT
sll1721_SF	GGCAATTACCAAATTCCCATG
sll1721_SR	ATTAGGGCAATGAGCTCAGC
slr1176_SF	TCGTTAAAATTTACGTCCTTACC
slr1176_SR	CAATACTTCCTTCTTGAAAACG
**D. Primers for *Synechocystis* colony PCR to verify self-replicating vector conjugation**	
VF2	TGCCACCTGACGTCTAAGAA
kivd^{S286T}_SR	TTGGCATTACCCACCCATTTC
**E. Primers for PCR amplification of genes**	
alsS_His_XbaI_F	TATATCTAGAATGCATCATCACCATCACCACGGTAGCGGAAGTTTGACCAAAGCCACTAAAGAAC
SpeI_PstI_R	CTGCAGCGGCCGCTACTAG
alsS_BglII_F	TATAAGATCTTTGACCAAAGCCACTAAAGAAC
kivd^{S286T}_Flag_XbaI_F	TATATCTAGAATGGACTACAAGGATGACGATGACAAGGGTAGCGGAAGTGGATCTATG
kivd^{S286T}_BglII_F	TATAAGATCTATGTACACCGTGGGAGATTAC
alsS_RBS_His_XbaI_F	TATATCTAGATAGTGGAGGTACTAGAATGCATCATCACCATCACCACGGTAGCGGAAGTGGATCTTTGACCAAAG
ilvD_BglII_F	TATAAGATCTCCCAAATATCGTAGTGCCAC
slr1192^{OP}_BglII_F	TATAAGATCTATTAAAGCCTACGCCGCCTTG
slr1192^{OP}_RBS_Flag_X_F	TATATCTAGATAGTGGAGGTACTAGAATGGACTACAAGGATGACGATGACAAGGGTAGCGGAAGTGGATCTATTA
ilvC_BglII_F	TATAAGATCTGCCAACTATTTTAACACCCTGAAC
ilvD_RBS_Flag_XbaI_F	TATATCTAGATAGTGGAGGTACTAGAATGGACTACAAGGATGACGATGACAAG
ilvC_Flag_XbaI_F	TATATCTAGAATGGACTACAAGGATGACGATGACAAG

Table S4. Expression quantification for all enzymes. The expression level of each protein is presented by the corresponding band intensity. The unit is intensity x mm.

(A)

Strain	Strep_Kivd^{S286T}	Flag_IlvD	Flag_Kivd^{S286T}	Flag_Slr1192^{OP}	His_AlsS
**HX0**	958.350	-	-	-	-
**HX5**	588.083	-		-	946.625
**HX7**	644.958	-	727.659	-	664.993
**HX8**	656.598	837.838	-	-	658.074
**HX9**	645.787	-	-	945.828	542.009

(B)

Strain	Strep_Kivd^{S286T}	Flag_IlvD	Flag_Kivd^{S286T}	Flag_IlvC	Flag_Slr1192^{OP}	His_AlsS
**HX17**	674.681	-	-	-	-	
**HX42**	1613.323	589.138	-	1109.120	1291.419	700.185
**HX43**	829.775	680.393	937.143	548.623	276.035	529.258
**HX16**	831.799	-	556.277	-	-	-

(C)

Strain	Strep_Kivd^{S286T}	Flag_Kivd^{S286T}
**HX0**	958.350	-
**HX1**	871.292	697.498
**HX2**	728.270	957.658
**HX3**	711.630	837.493

(D)

Strain	Strep_Kivd^{S286T}	Flag_Kivd^{S286T}	His_Kivd^{S286T}
**HX16**	831.799	556.277	-
**HX15**	858.370	728.398	769.510
**HX28**	664.545	879.828	-
**HX29**	535.807	796.797	686.077

**HX39**	1340.429	710.878	806.272
**HX44**	1422.122	730.630	608.706
**HX45**	1491.164	781.867	-
**HX46**	736.397	947.892	655.056
**HX47**	2204.934	789.543	911.881
